# Access to abortion and subsequent fertility preferences and reproductive outcomes in Nepal (2019–2024): a prospective cohort study

**DOI:** 10.1016/j.lansea.2026.100832

**Published:** 2026-09-03

**Authors:** Christina Misunas, Diana Greene Foster, Rachel Murro, Mahesh C. Puri, Corinne H. Rocca

**Affiliations:** a Department of Demography, University of California, Berkeley, Berkeley, CA, USA; b Advancing New Standards in Reproductive Health, Department of Obstetrics, Gynecology and Reproductive Sciences, University of California, San Francisco, Oakland, CA, USA; c Department of Epidemiology and Biostatistics, University of California, San Francisco, San Francisco, CA, USA; d Center for Research on Environment Health and Population Activities, Kathmandu, Nepal

**Keywords:** Abortion, Fertility preferences, Reproductive autonomy, Undesired birth, Unintended birth, Longitudinal

## Abstract

**Background:**

Access to abortion is central to people’s ability to act on their fertility preferences, yet little research has examined whether the ability to avoid having an unwanted birth shapes subsequent fertility outcomes or enables people to align future childbearing with their preferences. This study examines whether and how access to a desired abortion is associated with fertility preferences and reproductive outcomes over the subsequent years.

**Methods:**

We analyzed data from the *Nepal Turnaway Study*, a five-year prospective cohort study of women seeking abortion, comparing those who obtained an abortion with those who were denied and subsequently gave birth. We examined fertility preferences over time using mixed-effects multinomial logistic regression, time to subsequent pregnancy and birth using flexible parametric survival models, and cumulative births using mixed-effects Poisson regression. Finally, we assessed whether subsequent births were undesired prior to conception using mixed-effects logistic regression.

**Findings:**

Among 1742 participants seeking abortion, 1467 had an abortion or miscarried (the *no-birth-to-index-pregnancy* group) and 275 were denied and gave birth (the *gave-birth-to-index-pregnancy* group). Initially (at year one through year four), the *no-birth-to-index-pregnancy* group was more open to pregnancy than the *gave-birth-to-index-pregnancy* group; by year five, their fertility preferences converged. Four years after return to fecundability, the cumulative incidence of a subsequent pregnancy was 41.8% in the *no-birth-to-index-pregnancy* group and 18.3% in the *gave-birth-to-index-pregnancy* group (difference: 23.5 percentage points; 95% CI: 17.3–29.7), and of a subsequent birth was 30.8% and 10.0%, respectively (difference: 20.8 percentage points; 95% CI: 15.1–26.6). The predicted mean total number of births, including the index birth, per woman by year five was 1.04 for the *gave-birth-to-index-pregnancy* group and 0.37 for the *no-birth-to-index-pregnancy* group (difference: 0.67; 95% CI: 0.56–0.79). A smaller proportion of post-index births were undesired (before conception) in the *no-birth-to-index-pregnancy* group compared with the *gave-birth-to-index-pregnancy* group (35.1% vs. 84.5%; difference: 49.4 percentage points; 95% CI: 33.8–65.0; p = 0.0001).

**Interpretation:**

Access to abortion shapes subsequent fertility preferences and outcomes and can lead to greater alignment between future reproductive preferences and outcomes.

**Funding:**

This study was supported by the 10.13039/100000002National Institutes of Health and the 10.13039/100000008David and Lucile Packard Foundation.

**Research in context**

**Evidence before this study**

Research has shown that people seek abortions for many reasons, some of which may relate to their past or current childbearing and their future fertility desires. Fertility desires can also shift over time in response to changing life conditions, relationships, or aspirations. Evidence shows that reproductive experiences themselves, such as childbearing, can further reshape fertility preferences.

Evidence from the United States (US) *Turnaway Study* has shown that abortion access plays a critical role in shaping the timing, spacing, and intendedness of subsequent pregnancies. Yet, there is a limited understanding of how abortion access shapes fertility trajectories in low- and middle-income contexts. Research on this topic has been constrained both by a lack of rigorous, longitudinal, prospective data on fertility desires that captures how they evolve over time and by the absence of appropriate comparison groups who can serve as a credible counterfactual to those who receive abortions. Little is known about how abortion access influences future fertility preferences and the alignment between those intentions and subsequent reproductive outcomes, particularly in low- and middle-income countries.

**Added value of this study**

Data from the *Nepal Turnaway Study*, the first prospective cohort study outside the US to examine the impact of receiving vs. being denied a legal abortion, allow us to examine how fertility preferences and outcomes evolve based on whether one is able or unable to access a wanted abortion.

A distinctive strength of this study lies in its repeated measurement of women’s pregnancy preferences using the rigorously developed, prospective Desire to Avoid Pregnancy (DAP) scale, administered every six months. This approach allows for a nuanced examination of how fertility preferences shift over time as women’s lives, circumstances, and aspirations change. Indeed, we find that after seeking an abortion, women in the *no-birth-to-index-pregnancy* group were initially (at year one through year four) more open to pregnancy than those in the *gave-birth-to-index-pregnancy* group.

The longitudinal design of this study also made it possible to examine how access to abortion shapes both women’s subsequent fertility and the degree to which subsequent births are aligned with women’s evolving fertility preferences. We find that after returning to fecundability, women in the *no-birth-to-index-pregnancy* group experienced a shorter time to subsequent pregnancy and birth compared to women in the *gave-birth-to-index-pregnancy* group. Despite this, by year five, women in the *gave-birth-to-index-pregnancy* group had a higher cumulative number of births than those in the *no-birth-to-index-pregnancy* group, indicating a sustained difference in cumulative childbearing between study groups over the study period. Finally, post-index births to mothers in the *no-birth-to-index-pregnancy* group were more likely to reflect their fertility preferences before conception, compared with births to those in the *gave-birth-to-index-pregnancy* group.

**Implications of all the available evidence**

Consistent with evidence from the US, our findings suggest that obtaining a desired abortion did not preclude Nepali women from becoming pregnant or giving birth in the future; instead, it was associated with childbearing that more closely aligned with women’s fertility preferences as they changed over time.

By allowing women to act on their fertility preferences both at the time of abortion seeking and in the years that follow, abortion access may support both the prevention of undesired pregnancies and the realization of childbearing when and under the circumstances they choose. Providing this evidence within the context of Nepal offers important insights into how abortion access can influence fertility trajectories in a lower-income setting undergoing a fertility transition where abortion is legal but significant barriers to access persist.

## Introduction

Over the past 25 years, Nepal has undergone a fertility transition, with the total fertility rate falling from 4.6 children per woman in 1996 to 2.1 in 2022, and the median birth interval increasing from 31.8 months in 2001 to 40.7 months in 2022.^1^ Changes in fertility intentions have occurred in parallel. Women’s average ideal number of children declined from 2.9 children per woman in 1996 to 2.0 in 2022.^1^ Among all pregnancies, the percentage of wanted births or current pregnancies increased from 62% in 1996 to 79% in 2022, while mistimed births decreased from 19% to 15% and unwanted births declined from 18% to 6%.^1^

Despite these substantial shifts, gaps between women’s childbearing desires and births remain. The total fertility rate among women aged 15–49 in Nepal exceeds the wanted fertility rate by 0.4 children.^1^ Being low income and having lower levels of education are associated with a higher likelihood of exceeding one’s ideal family size.^1^ This gap between desired and achieved fertility, particularly among disadvantaged groups, suggests that many women continue to face constraints on their reproductive autonomy. In addition to infringing on the human right of individuals and couples to “decide freely the number and spacing of their children,”^2^ undesired births may also compromise the health and well-being of mothers and children.^3^, ^4^, ^5^, ^6^

Abortion can be an important tool for achieving a smaller desired family size, delaying childbearing, and increasing birth spacing. The decision to terminate a pregnancy may also reflect women’s assessments of their current circumstances and be distinct from their longer-term fertility goals, particularly when those goals are uncertain.^7^^,^^8^ Ultimately, the ability to exercise autonomy over one’s reproductive trajectory and realize one’s fertility goals can depend on access to safe abortion services.

In Nepal, abortion was legalized in 2002 and is broadly permitted upon request up to 12 weeks of gestation.^9^ The 2018 Safe Motherhood and Reproductive Health Rights Act broadened legal eligibility, permitting termination up to 28 weeks in cases of rape or incest, for women with HIV or other incurable diseases, or for certain mental health conditions.^10^ Since legalization, the Nepali Ministry of Health and Population has expanded access by training and certifying abortion providers and equipping and certifying health facilities to provide safe abortion services.^11^^,^^12^ Services are available through government-approved health facilities, including public hospitals, primary healthcare centers, health posts, and selected non-governmental organizations. Provision depends on length of gestation and provider training, with first-trimester services provided by trained physicians and mid-level providers and second-trimester services restricted to pre-approved hospitals and specialized physicians.^11^

Despite legal protections and expanded service delivery, barriers to accessing safe abortion persist, and abortions outside the formal system continue to occur.^13^, ^14^, ^15^ Limited awareness of abortion laws and service availability among healthcare providers^16^ and women^17^ remain significant barriers to accessing safe abortion.^15^ These challenges disproportionately affect the poorest, most disadvantaged, and most geographically isolated women.^18^ Findings from the *Nepal Turnaway Study* also highlighted these inequities: women with lower socioeconomic status, including those who were younger, less educated, and less wealthy, were more likely to present later for abortion, be turned away, and continue the pregnancy after abortion denial.^19^

The broader literature has shown that people may seek abortions for many complex and overlapping reasons, some of which might be tied to their past or current childbearing responsibilities and their future fertility goals.^7^^,^^8^ For some, the decision to terminate a pregnancy reflects that they do not want any (additional) children. Others might use abortion to delay childbearing, for example, when their current life circumstances are not amenable to raising a child. The needs of existing children can also shape the decision to terminate a pregnancy.^7^

Understanding the role of abortion in individuals’ future fertility preferences is important for evaluating how access to abortion (or lack thereof) can influence longer-term reproductive trajectories. However, uncertainty around fertility expectations is common^20^ and fertility preferences may shift in response to changes in health, partnerships, or economic conditions. Evidence also suggests that fertility preferences can both influence reproductive outcomes and evolve in response to lived reproductive experiences.^20^, ^21^, ^22^, ^23^ In the context of abortion-seeking, these preferences may evolve further; for example, being denied access to an abortion and giving birth may prompt individuals to reassess their future childbearing desires. The dynamic nature of fertility preferences highlights the importance of ensuring that individuals can make reproductive decisions that reflect their preferences not only in the moment, but as their lives, circumstances, and goals change over time.

Additionally, access to abortion may have measurable effects on the timing, spacing, and intendedness of subsequent pregnancies. Studies in the United States have found that, compared to women who gave birth after being denied abortions, those who received abortions and delayed childbearing were less likely to have short birth intervals,^8^ which can have important consequences for the health and well-being of their pregnancies.^24^ Women who received abortions were also more likely to have subsequent intended pregnancies than those who gave birth after being denied abortions.^25^ However, most existing evidence on the relationship between abortion access and subsequent fertility comes from high-income settings, with far less known about these dynamics in low- and middle-income contexts.

The *Nepal Turnaway Study*, a prospective cohort study that examined the impact of receiving vs. being denied a legal abortion, allows us to assess how fertility preferences and outcomes can evolve in response to lived reproductive experiences, specifically whether or not one is forced to carry an unwanted pregnancy to term. Examining these dynamics in Nepal is particularly valuable, as it provides insight into how abortion access shapes fertility trajectories in a lower-income setting undergoing a fertility transition, where abortion is legal but implementation challenges persist.

This paper aims to answer the following research questions: What are women’s lifetime fertility desires when they seek abortions, and how do their fertility preferences evolve based on whether they are able to access a desired abortion or are denied and give birth? How does access to abortion influence women’s pregnancy and childbearing trajectories in the years following abortion seeking? To what extent do subsequent births to women who access abortion reflect their fertility preferences?

## Methods

The *Nepal Turnaway Study* is a nationwide prospective cohort study conducted among women who sought abortions at facilities in Nepal for pregnancies they did not want to carry to term. The study was designed to examine the impact of receiving vs. being denied a legal abortion on women’s mental and physical health, fertility outcomes and preferences, as well as the broader social and economic consequences for both women and their children over a five-year period.

Eligible participants were women aged fifteen or older who lived in Nepal, were pregnant, and were seeking abortion care. After the first month of recruitment, eligibility was restricted to women presenting at or beyond ten weeks’ gestation or who were uncertain of their gestational duration to ensure sufficient representation of those more likely to be denied care. No other exclusion criteria were applied.

Between April 2019 and December 2020, women aged 15–45 years were recruited from 22 health facilities across Nepal’s seven provinces. Facilities were randomly selected with probability proportional to client volume from a national sampling frame of government-certified facilities that provided at least 60 safe abortions during 2016–2017 through inpatient, outpatient, or both types of care. All selected facilities agreed to participate. Recruitment began at 14 public and private/non-profit facilities, with one of each type per province. In February 2020, seven facilities with low volumes of eligible participants were replaced with facilities of the same type and within the same province. One additional site was added in June 2020 using the same sampling strategy.

At each facility, staff screened potential participants for eligibility and documented gestational duration based on last menstrual period. A trained research staff member confirmed eligibility, obtained informed consent in writing or by thumbprint, and administered the baseline survey in a private room using a tablet before participants learned whether they would receive abortion services or be denied.

Follow-up interviews were conducted in participants’ preferred language at their homes or another location of their choosing six weeks after seeking an abortion and every six months thereafter for five years. Interviews lasted approximately 45 min. Participants received NRs 500 (approximately USD 4) for each interview. More details on study design and procedures are provided in Murro et al.^26^

The outcome of the index pregnancy, ascertained during follow-up, served as the primary exposure and basis for the study groups. The *gave-birth-to-index-pregnancy* group comprised participants who were denied an abortion and subsequently gave birth. The *no-birth-to-index-pregnancy* group comprised participants who received an abortion on the day of recruitment or were no longer pregnant by the six-week follow-up because they had obtained an abortion later or elsewhere after an initial denial. This group also included a small number of participants who reported a miscarriage or no longer being pregnant without specifying how the index pregnancy ended.

We assessed the following outcomes: lifetime fertility desires at the time of abortion seeking, fertility preferences over time, time to subsequent pregnancy and birth, cumulative births over the five-year period, and whether subsequent births aligned with mothers’ fertility preferences.

Lifetime fertility desires were assessed at baseline before participants learned whether they would receive an abortion. Respondents were asked: “Would you like to have (more) children (than you have now)?” “How many (more) children would you like to have?” and “When would you like to have (another) child?”

At each follow-up interview, respondents who were not pregnant completed the 14-item Desire to Avoid Pregnancy (DAP) scale. The DAP scale is a psychometrically validated, unidimensional measure covering cognitive desires and preferences, affective feelings and attitudes, and anticipated practical consequences.^27^ Participants rated their agreement with statements about becoming pregnant in the next three months or having a baby within one year on a 0–4 Likert scale. Item scores were averaged, with higher scores indicating greater desire to avoid pregnancy. Following expert guidance,^28^ we created a four-category outcome variable: strong desire to avoid pregnancy (DAP > 2.5), moderate desire to avoid pregnancy (DAP > 1.5 to 2.5), open to pregnancy (DAP ≤ 1.5), and currently pregnant (no DAP score recorded).

To identify pregnancies and births over the five-year observation period, including from the index pregnancy, participants were asked at each survey whether they were pregnant or had been pregnant in the past six months, and whether any pregnancy had ended in birth. For pregnancies ending in birth, we estimated conception as occurring nine months before the reported birth date. If a participant reported being pregnant within the past six months but was no longer pregnant and the pregnancy had not ended in birth, we estimated conception as occurring three months before the survey.

For women denied an abortion who gave birth, cumulative births over the five-year period included both the index birth and any subsequent births. *Births* refer to live births; counts do not account for whether the child survived after birth.

To assess whether a subsequent birth was aligned with the mother’s fertility preferences, we linked each birth to the mother’s pre-pregnancy DAP score. Specifically, a birth was classified as undesired if the DAP score recorded at the interview prior to the estimated date of conception was >2.5, meaning the mother had expressed a strong desire to avoid pregnancy.

### Statistical analyses

We described sample characteristics and lifetime fertility desires at baseline and assessed differences by study group using logistic and multinomial regression models (for binary and categorical variables, respectively), accounting for facility-level clustering with robust standard errors. We used propensity score overlap weights to adjust for baseline confounding and to balance covariate distributions between study groups. Propensity scores were estimated from a multivariable logistic regression model predicting study group membership (*no-birth-to-index-pregnancy* vs. *gave-birth-to-index-pregnancy*) using baseline sociodemographic characteristics (age, marital status, education, employment, parity, income), index pregnancy characteristics (gestational duration, abortion seeking due to fetal anomaly or sex selection, retrospective pregnancy intendedness), clinic characteristics (distance from participant home, public/private), medical history (anxiety/depression, chronic pain, intimate partner violence), measures of empowerment, and husbands’ drinking behavior. Details on weight development are provided in Murro et al.^26^ To confirm baseline comparability, we assessed differences between study groups before and after applying the weights.

All analyses applied these weights and used cluster-robust standard errors at the facility level. For each analysis, we used a time frame appropriate for the outcome and research question.

To examine the association between study group and fertility preferences after abortion seeking, we estimated a mixed-effects multinomial logistic regression model using the four-category DAP scale variable as the outcome. We modeled fertility preferences starting one year after abortion seeking, when women in the *gave-birth-to-index-pregnancy* group were no longer pregnant and therefore could become pregnant, through year five. The model included study group, time since abortion seeking (in years), and their interaction to assess change over time and whether trajectories differed by study group. Individual-level random intercepts were included to account for within-person correlation across repeated observations. Marginal predicted probabilities were obtained based on these models.

We modeled time to the next pregnancy and, separately, birth using flexible parametric survival models to accommodate non-proportional hazards. Models were fit using the *stpm2* module in Stata and included a time-varying study group effect, allowing each group’s hazard to vary over time.^29^ We estimated standardized cumulative incidence, between-group differences, and restricted mean survival time (RMST). Time was measured from entry into the risk period, defined as one month after abortion seeking for the *no-birth-to-index-pregnancy* group and three months after the index birth for the *gave-birth-to-index-pregnancy* group to account for reduced likelihood of conception during the postpartum period. A sensitivity analysis used six-month postpartum entry for the *gave-birth-to-index-pregnancy* group. Follow-up was administratively censored at 1888 days (∼5.2 years) for the *no-birth-to-index-pregnancy* group and 1500 days (∼4.1 years) for the *gave-birth-to-index-pregnancy* group. Accordingly, we compared cumulative incidence and RMST at four years after entry into the risk period, the longest interval for which both groups had follow-up data.

To estimate cumulative births by year five, we used a mixed-effects Poisson regression model, which provided interval-specific birth rates from recruitment (time 0) through year five. The model included study group, time since abortion seeking, and their interaction as well as individual-level random intercepts. To capture non-linear changes in fertility over time, we tested quadratic and cubic time terms; we retained the cubic term based on improved model fit using the likelihood ratio test. The model included a log-transformed offset for interval length (in years) to account for variation in exposure time. A negative binomial model produced substantively similar results without improving model fit, so we retained the Poisson specification. We estimated predicted birth counts per 0.5-year interval using population-average predictions via the *margins* command and summed these counts to calculate cumulative births by year five.

Finally, we used mixed-effects logistic regression to examine the association between study group and the likelihood that a subsequent birth was undesired prior to conception. The analysis included all post-index births for which a pre-pregnancy DAP score was available. The model included study group and time since abortion seeking as well as a random intercept for each woman to account for multiple births per individual. Marginal predicted probabilities were estimated from this model.

We conducted sensitivity analyses using alternative study group definitions, excluding women with presumed miscarriages from the *no-birth-to-index-pregnancy* group.

Across models, the *gave-birth-to-index-pregnancy* group was used as the reference group. Statistical significance was defined as p < 0.05. Analyses were conducted using Stata version 17.

### Ethics statement

This study is registered with ClinicalTrials.gov, number NCT03930576. The study funders had no role in study design, data collection, data analysis, interpretation, decision to publish, or preparation of the manuscript.

Ethical review and approval of this study were provided by The University of California, San Francisco Human Research Protection Program (18–25863, initial approval on April 15, 2019) and the Ethics Committee of the Nepal Health Research Council (704/2018, initial approval on December 26, 2018).

## Results

A total of 1841 women aged 15–45 completed baseline interviews; 90 (5%) were lost to follow-up after baseline, and nine (0.5%) were excluded after determining they had not been pregnant when seeking an abortion. The final analytic sample included 1742 women with a mean follow-up of 56 months (SD = 12.4).

Among the analytic sample, 1467 women either received an abortion on the day that they were recruited (n = 890) or were no longer pregnant at the first six-week follow-up interview because they had an abortion (n = 536) or miscarried (n = 41) subsequent to baseline (the *no-birth-to-index-pregnancy* group); 275 were denied and gave birth (the *gave-birth-to-index-pregnancy* group).

By the final interview, 62% (n = 1073) were retained, 27% (n = 478) were administratively censored after 48 months because the study ended before they completed five years of follow-up, and 11% (n = 191) were lost to follow-up. Attrition did not differ by study group. Administrative censoring was related to recruitment timing and was accounted for through the use of propensity scores, which incorporated baseline characteristics. Final interviews occurred 4.75 to 5.67 years after initial abortion-seeking, with a median follow-up of 5.3 years. Additional details are available in Murro et al.^26^

Table 1 presents demographic characteristics and lifetime fertility desires of the overall sample and by study group at baseline. Most participants were married (97.2%). Overall, 13.3% had no living children, 29.8% had one, 35.7% had two, and 21.3% had three or more. In the unweighted sample, women in the *gave-birth-to-index-pregnancy* group were significantly younger (≤24 years old: 40.0% vs. 32.0%; p = 0.023), more likely to belong to the Dalit/other caste (24.5% vs. 13.1%; p < 0.0001), and more likely to report income inadequacy (39.0% vs. 24.4%; p = 0.019) than those in the *no-birth-to-index-pregnancy* group. They were more likely to have completed only primary schooling (23.4% vs. 13.9%; p = 0.0002) and less likely to have completed secondary schooling (54.6% vs. 63.0%; p = 0.010) or higher (4.0% vs. 7.4%; p = 0.040). Compared to women in the *no-birth-to-index-pregnancy group*, those in the *gave-birth-to-index-pregnancy* group were also significantly more likely to have a husband who drinks daily, experience intimate partner violence, lack decision-making power in the household, and have limited mobility outside the home (not shown).

**Table 1:** Baseline demographic characteristics and lifetime fertility desires, overall and by study group.

	Unweighted				Weighted		
	Total (n = 1742)	No-birth-to-index-pregnancy (n = 1467)	Gave-birth-to-index-pregnancy (n = 275)	p-value	No-birth-to-index-pregnancy	Gave-birth-to-index-pregnancy	p-value
Sociodemographic characteristics							
Age in years							
≤24	579 (33.2)	469 (32.0)	110 (40.0)	0.023	(38.6)	(38.6)	0.99
25–29	504 (28.9)	415 (28.3)	89 (32.4)	0.07	(32.6)	(32.6)	0.99
30–34	380 (21.8)	332 (22.6)	48 (17.5)	0.047	(17.8)	(17.8)	0.99
35+	279 (16.0)	251 (17.1)	28 (10.2)	<0.0001	(10.9)	(10.9)	0.99
Caste							
Brahmin, Chhetri, or Thakuri	683 (39.4)	594 (40.6)	89 (32.6)	0.08	(34.5)	(34.2)	0.93
Hill Janajati	415 (23.9)	346 (23.7)	69 (25.3)	0.78	(24.9)	(25.1)	0.98
Dalit or other	259 (14.9)	192 (13.1)	67 (24.5)	<0.0001	(21.6)	(21.7)	0.97
Terai Janajati	378 (21.8)	330 (22.6)	48 (17.6)	0.20	(19.0)	(19.1)	0.98
Marital status							
Not married	48 (2.8)	42 (2.9)	6 (2.2)	0.58	(2.6)	(2.6)	0.99
Married	1694 (97.2)	1425 (97.1)	269 (97.8)		(97.4)	(97.4)	
Number of living children							
None	231 (13.3)	200 (13.6)	31 (11.3)	0.41	(12.1)	(12.1)	0.99
One	520 (29.8)	438 (29.9)	82 (29.6)	0.99	(30.0)	(30.0)	0.99
Two	621 (35.7)	530 (36.1)	91 (33.2)	0.41	(33.8)	(33.8)	0.99
Three or more	370 (21.3)	299 (20.4)	71 (25.9)	0.036	(24.1)	(24.1)	0.99
Educational attainment							
None or some formal schooling	279 (16.1)	230 (15.7)	49 (17.9)	0.27	(17.4)	(17.6)	0.90
Primary	267 (15.4)	203 (13.9)	64 (23.4)	0.0002	(21.1)	(21.1)	0.98
Secondary	1069 (61.6)	920 (63.0)	149 (54.6)	0.010	(56.9)	(56.6)	0.93
Higher	119 (6.9)	108 (7.4)	11 (4.0)	0.040	(4.7)	(4.7)	0.99
Household income adequacy							
Not adequate	463 (26.7)	357 (24.4)	106 (39.0)	0.019	(34.7)	(35.0)	0.96
Adequate	1228 (70.9)	1066 (73.0)	162 (59.6)	0.034	(63.7)	(63.4)	0.95
More than adequate	42 (2.4)	38 (2.6)	4 (1.5)	0.35	(1.5)	(1.5)	0.99
Employed							
No	800 (46.1)	658 (45.0)	142 (52.0)	0.19	(51.1)	(50.8)	0.96
Yes	935 (53.9)	804 (55.0)	131 (48.0)		(48.9)	(49.2)	
Index pregnancy characteristics							
Gestational duration							
At or below ten weeks	648 (37.2)	613 (41.8)	35 (12.7)	<0.0001	(16.3)	(16.3)	0.99
Above ten weeks	635 (36.5)	486 (33.1)	149 (54.2)	0.002	(51.0)	(51.0)	0.99
Don’t know	459 (26.3)	368 (25.1)	91 (33.1)	0.26	(32.6)	(32.6)	0.99
Seeking abortion due to fetal anomaly							
No or don’t know	1561 (89.6)	1293 (88.1)	268 (97.5)	0.0002	(96.6)	(96.6)	0.99
Yes	181 (10.4)	174 (11.9)	7 (2.5)		(3.4)	(3.4)	
Seeking abortion due to sex selection							
No or don’t know	1575 (90.4)	1351 (92.1)	224 (81.5)	<0.0001	(83.5)	(83.5)	0.99
Yes	167 (9.6)	116 (7.9)	51 (18.5)		(16.5)	(16.5)	
LMUP score, mean (SD)	3.7 (2.7)	3.7 (2.7)	3.8 (2.6)	0.07	3.7 (2.6)	3.7 (2.6)	0.65
Lifetime fertility desires							
Desired number of (additional) children							
None	1066 (61.4)	899 (61.5)	167 (60.9)	0.91	(58.7)	(60.9)	0.63
One	491 (28.3)	405 (27.7)	86 (31.4)	0.41	(32.7)	(31.2)	0.74
Two	133 (7.7)	117 (8.0)	16 (5.8)	0.16	(6.0)	(6.2)	0.85
Three or more	2 (0.1)	1 (0.1)	1 (0.4)	0.40	(0.1)	(0.4)	0.55
Don’t know or refused	44 (2.5)	40 (2.7)	4 (1.5)	0.16	(2.5)	(1.4)	0.17
Desired timing of (additional) children							
Never	1066 (61.4)	899 (61.5)	167 (60.9)	0.91	(58.7)	(60.9)	0.63
Within one year	43 (2.5)	34 (2.3)	9 (3.3)	0.53	(3.1)	(3.3)	0.95
In one to two years	186 (10.7)	159 (10.9)	27 (9.9)	0.80	(11.1)	(9.8)	0.72
In three to five years	210 (12.1)	177 (12.1)	33 (12.0)	0.98	(12.8)	(11.6)	0.68
In five or more years	120 (6.9)	97 (6.6)	23 (8.4)	0.37	(8.8)	(8.8)	0.99
When God wants	45 (2.6)	39 (2.7)	6 (2.2)	0.70	(2.2)	(2.2)	0.96
Don’t know or refused	66 (3.8)	57 (3.9)	9 (3.3)	0.66	(3.2)	(3.5)	0.86

**Notes:** Unweighted data are presented as n (column %) unless otherwise noted. Weighted columns show propensity-score weighted percentages; counts are not shown because weighted results apply to an artificially balanced sample. For binary variables, p-values were obtained from logistic regression models comparing study groups. For categorical variables, p-values represent category-specific average marginal differences in predicted probabilities between study groups, estimated after multinomial logistic regression using *margins*. All models used facility-clustered robust standard errors. Weighted models incorporated propensity-score overlap weights derived from models predicting study group membership. LMUP = London Measure of Unplanned Pregnancy; SD = standard deviation. LMUP scores are generally interpreted as unplanned (0–3), ambivalent (4–9), or planned (10–12).

Index pregnancy characteristics also differed between groups (Table 1). Women in the *gave-birth-to-index-pregnancy* group were more likely to present for care after ten weeks’ gestation (54.2% vs. 33.1%; p = 0.002) and to seek abortion due to sex selection (18.5% vs. 7.9%; p < 0.0001), but less likely to seek abortion due to fetal anomaly (2.5% vs. 11.9%; p = 0.0002). However, mean London Measure of Unplanned Pregnancy (LMUP) scores indicated similarly low levels of planning and intendedness for the index pregnancy in both groups.

After propensity score weighting, baseline covariates were well balanced between groups (Table 1).

At the time of seeking an abortion, women expressed a range of lifetime fertility desires: 61.4% wanted no future children, 28.3% wanted one, 7.7% wanted two, 0.1% wanted three or more, and 2.5% were unsure or refused to answer (Table 1). Regarding the timing of future childbearing, 2.5% of all women wanted a child within a year; 10.7%, in one to two years; 12.1%, in three to five years; and 6.9%, in five or more years. Lifetime fertility desires at baseline did not differ significantly between the study groups (Table 1).

The DAP scale demonstrated high internal consistency (Cronbach’s alpha: 0.97). Fig. 1 presents model-predicted probabilities of pregnancy preferences and pregnancy status at one year after seeking an abortion through year five, by study group. At one year after seeking an abortion, 67.4% of women in the *no-birth-to-index-pregnancy* group expressed a strong desire to avoid pregnancy, 10.3% expressed a moderate desire to avoid pregnancy, 13.6% were open to pregnancy, and 8.8% were currently pregnant (Fig. 1, Supplementary Table S1). Among those in the *gave-birth-to-index-pregnancy* group, 94.9% expressed a strong desire to avoid pregnancy, 3.3% expressed a moderate desire to avoid pregnancy, 0.8% were open to pregnancy, and 1.1% were currently pregnant (Fig. 1, Supplementary Table S1).

**Fig. 1:**
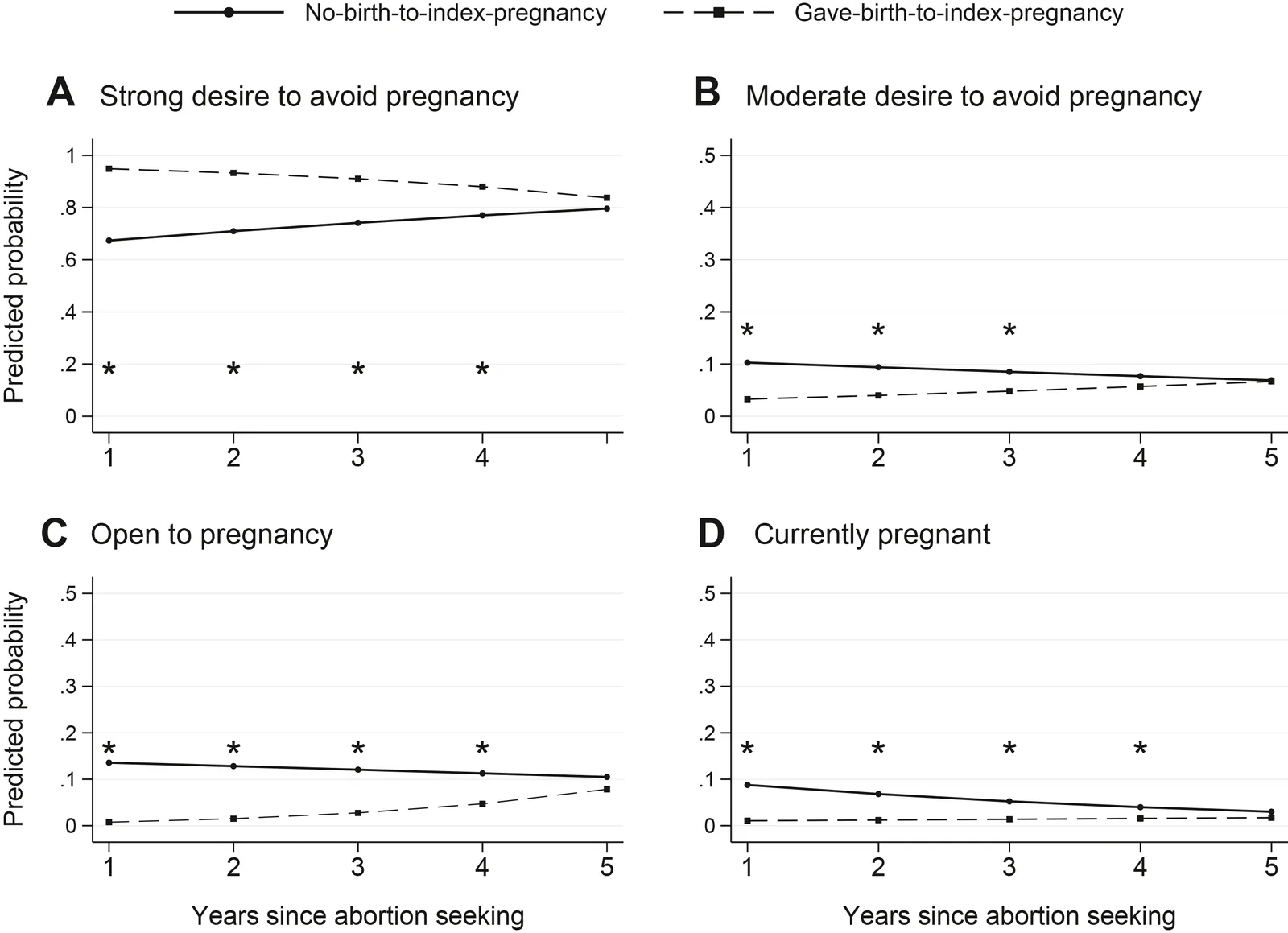
**Model-predicted probabilities of pregnancy preferences and pregnancy status after abortion seeking, by study group.** Values represent marginal predicted probabilities from a mixed-effects multinomial logistic regression used to model changes in pregnancy preferences and pregnancy status from one through five years after abortion seeking. The categorical outcome comprised four categories, shown in panels A–D: (A) strong desire to avoid pregnancy (DAP > 2.5), (B) moderate desire to avoid pregnancy (DAP > 1.5 to 2.5), (C) openness to pregnancy (DAP ≤ 1.5), and (D) currently pregnant (no DAP scale score recorded). The model included study group, time since abortion seeking (in years), and their interaction, with individual-level random intercepts to account for within-person correlation across repeated observations. Cluster-robust standard errors at the facility level and propensity score weights were applied. DAP = Desire to Avoid Pregnancy scale. ∗Indicates a significant risk difference (p < 0.05) between the study groups at each time point.

Among the *no-birth-to-index-pregnancy* group, adjusted relative risk ratios for being open to pregnancy, moderately avoidant of pregnancy, and currently pregnant (vs. strongly avoidant of pregnancy) declined modestly over time (ARRRs = 0.86, 0.84, and 0.72 per year, respectively; Supplementary Table S2). Conversely, in the *gave-birth-to-index-pregnancy* group, the relative risk ratios for being open to pregnancy, moderately avoidant of pregnancy, and currently pregnant (vs. strongly avoidant) all increased over time (ARRRs = 2.43, 1.29, and 1.14 per year, respectively); however, only the increase in openness to pregnancy was statistically significant (Supplementary Table S2). By year five, differences in pregnancy preferences and pregnancy status between groups were no longer statistically significant (Fig. 1, Supplementary Table S1).

Cumulative incidence curves and RMST demonstrated distinct trajectories for the study groups, with the *no-birth-to-index-pregnancy* group experiencing a shorter time to subsequent pregnancy and birth (Fig. 2). Four years after entry into the risk period, the model-predicted cumulative incidence of a subsequent pregnancy was 41.8% (95% CI: 38.1–45.3) for the *no-birth-to-index-pregnancy* group and 18.3% (95% CI: 13.2–23.2) for the *gave-birth-to-index-pregnancy* group (difference: 23.5 percentage points; 95% CI: 17.3–29.7) (Fig. 2, Supplementary Table S3). The RMST indicated that, on average, the *no-birth-to-index-pregnancy* group did not experience a subsequent pregnancy for 2.97 years (95% CI: 2.88–3.08) compared with 3.65 years (95% CI: 3.53–3.76) for the *gave-birth-to-index-pregnancy* group (difference: 0.67; 95% CI: 0.52–0.82) (not shown).

**Fig. 2:**
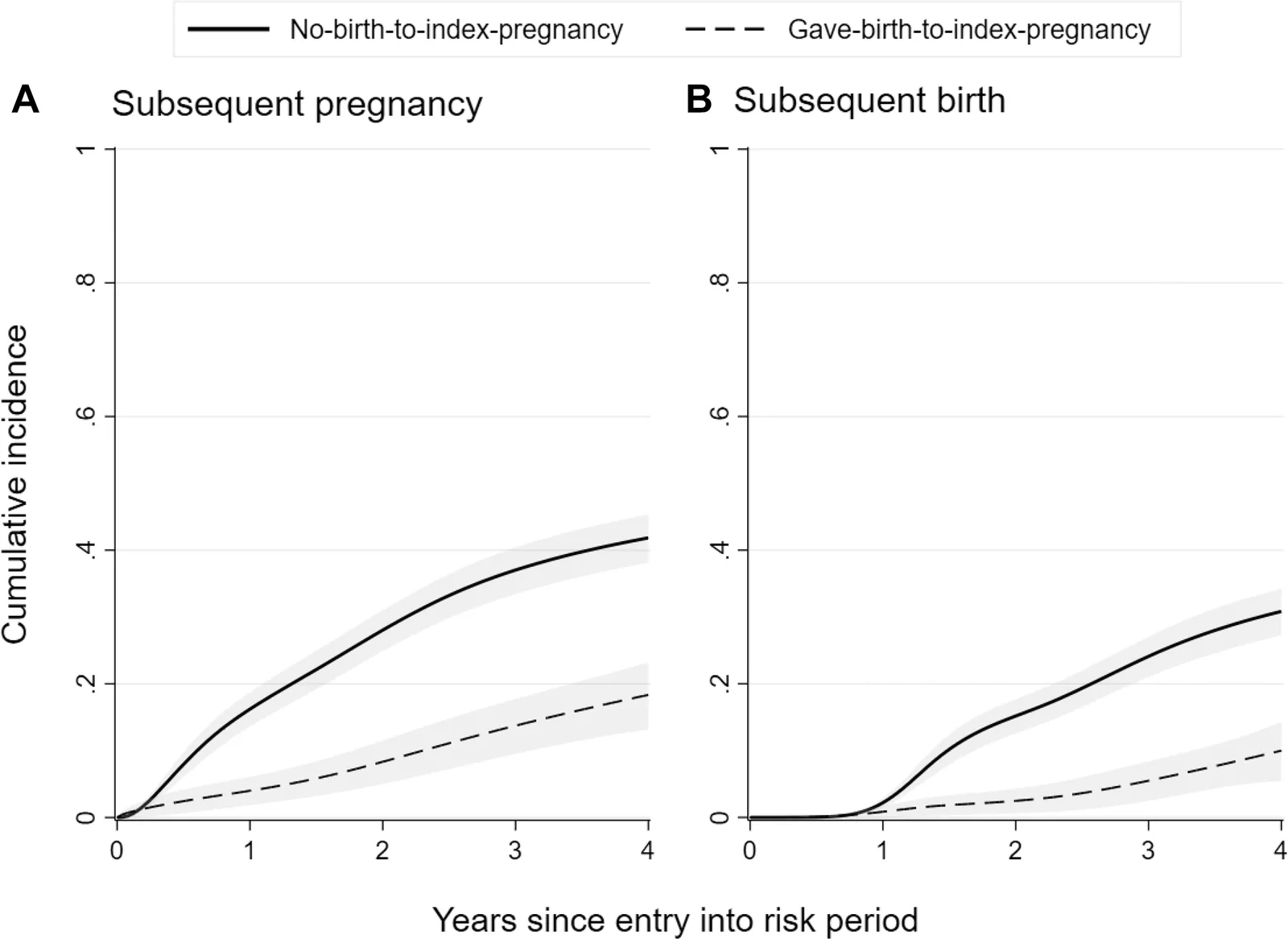
**Model-predicted cumulative incidence of(A)subsequent pregnancy and(B) subsequentbirth, by study group.** Flexible parametric survival models were used to model time to the next (non-index) pregnancy and birth. Models included a time-varying study group effect, which allowed the hazard ratio between groups to vary over time, as well as propensity score weights and cluster-robust standard errors at the facility level. Solid and dashed lines show model-predicted cumulative incidence, and shaded areas show 95% confidence intervals. Among the *no-birth-to-index-pregnancy* group (n = 1467), 587 women experienced at least one subsequent pregnancy and 420 had at least one subsequent birth during follow-up. Among the *gave-birth-to-index-pregnancy* group (n = 275), 47 women experienced at least one subsequent pregnancy and 22 had at least one subsequent birth during follow-up. For the *no-birth-to-index-pregnancy* group, entry into the risk period was set to one month after abortion seeking at the baseline interview. For the *gave-birth-to-index-pregnancy* group, entry into the risk period was set to three months after the index birth to account for the postpartum period during which conception was unlikely. Follow-up was administratively censored at 1888 days (∼5.2 years) for the no-birth-to-index-pregnancy group and 1500 days (∼4.1 years) for the *gave-birth-to-index-pregnancy* group. To enable comparability across study groups, plotted curves were restricted to four years since entry into the risk period, when both groups had sufficient follow-up data.

Four-year cumulative incidence of a subsequent birth was 30.8% (95% CI: 27.3–34.2) for the *no-birth-to-index-pregnancy* group and 10.0% (95% CI: 5.4–14.3) for the *gave-birth-to-index-pregnancy* group (difference: 20.8 percentage points; 95% CI: 15.1–26.6) (Fig. 2, Supplementary Table S3). As measured by the RMST, on average, women in the *no-birth-to-index-pregnancy* group did not experience a subsequent birth for 3.43 years (95% CI: 3.36–3.50) compared with 3.87 years (95% CI: 3.80–3.93) in the *gave-birth-to-index-pregnancy* group (difference: 0.44; 95% CI: 0.34–0.54) (not shown).

Results were consistent when entry into the risk period was defined as six months after the index birth (Supplementary Table S4).

Over the five-year period, among women in the *no-birth-to-index-pregnancy* group, 71% had no births, 26% had one birth, and 3% had two births. Among women in the *gave-birth-to-index-pregnancy* group, 92% had one birth (the index birth), 7% had two births (the index birth and one subsequent birth), and <1% had three births (the index birth and two subsequent births) (not shown).

Table 2 presents predicted birth counts (per 0.5-year interval), estimated from a mixed-effects Poisson regression model (Supplementary Table S5). The estimated cumulative number of births per woman over the five-year period was 1.04 (95% CI: 0.97–1.12) in the *gave-birth-to-index-pregnancy* group and 0.37 (95% CI: 0.29–0.45) in the *no-birth-to-index-pregnancy* group, a difference of 0.67 births per woman (95% CI: 0.56–0.79, p < 0.0001).

**Table 2:** Model-predicted birth counts per woman over the five-year period, cumulatively and per 0.5-year intervals, by study group.

	No-birth-to-index-pregnancy			Gave-birth-to-index-pregnancy			Predicted difference in births between study groups			
	Predicted births	Lower CI	Upper CI	Predicted births	Lower CI	Upper CI	Predicted difference	Lower CI	Upper CI	p-value
0–5 (cumulative)	0.37	0.29	0.45	1.04	0.97	1.12	0.67	0.56	0.79	<0.0001
Interval (years)										
0–0.5	0.01	0.01	0.02	0.69	0.59	0.81	0.68	0.57	0.79	<0.0001
0.5–1	0.03	0.02	0.05	0.25	0.21	0.29	0.22	0.17	0.26	<0.0001
1–1.5	0.05	0.04	0.07	0.07	0.05	0.10	0.02	−0.01	0.04	0.25
1.5–2	0.06	0.05	0.08	0.02	0.01	0.05	−0.05	−0.07	−0.02	<0.0001
2–2.5	0.06	0.05	0.07	0.005	0.0007	0.03	−0.06	−0.07	−0.04	<0.0001
2.5–3	0.05	0.04	0.06	0.002	0.0001	0.02	−0.05	−0.06	−0.04	<0.0001
3–3.5	0.04	0.03	0.05	0.001	0.00004	0.02	−0.04	−0.04	−0.03	<0.0001
3.5–4	0.03	0.02	0.03	0.001	0.00003	0.02	−0.03	−0.03	−0.02	<0.0001
4–4.5	0.02	0.01	0.03	0.002	0.0001	0.03	−0.02	−0.03	−0.01	<0.0001
4.5–5	0.02	0.01	0.03	0.01	0.001	0.05	−0.01	−0.02	0.01	0.37

**Notes:** The number of children born over the five-year period was modeled using mixed-effects Poisson regression, with study group, time since abortion seeking (in years), and their interaction as predictors. Time was modeled using cubic polynomial terms and their interactions with study group to allow for non-linear fertility trajectories. Person-time between interviews (in years) was included as an offset to estimate birth rates. The model included individual-level random intercepts, propensity score weights, and facility-level cluster-robust standard errors. Interval-specific predicted birth counts were obtained by multiplying estimated birth rates by the interval length (0.5 years); cumulative birth counts were calculated by summing predicted counts across all intervals while accounting for their covariance. CI = confidence interval.

Among all subsequent births (n = 481), 458 occurred among 420 women in the *no-birth-to-index-pregnancy* group and 23 among 22 women in the *gave-birth-to-index-pregnancy* group. Pre-pregnancy DAP scores were available for 98% (n = 471) of subsequent births.

Subsequent births in the *no-birth-to-index-pregnancy* group had significantly lower odds of being undesired prior to conception than subsequent births in the *gave-birth-to-index-pregnancy* group (AOR = 0.10; 95% CI: 0.03–0.32; p = 0.0001; Table 3). Model-predicted probabilities indicated that 35.1% (95% CI: 26.4–43.8) of subsequent births in the *no-birth-to-index-pregnancy* group were undesired (before conception), compared with 84.5% (95% CI: 69.0–99.9) in the *gave-birth-to-index-pregnancy* group, a difference of 49.4 percentage points (95% CI: 33.8–65.0; p < 0.0001).

**Table 3:** Mixed-effects logistic regression estimates and model-predicted probabilities that subsequent births were undesired prior to conception, by study group.

Panel A: Adjusted odds that a subsequent birth was undesired (before conception).								
Predictor	Unweighted model				Weighted model			
	AOR	Lower CI	Upper CI	p-value	AOR	Lower CI	Upper CI	p-value
Gave-birth-to-index-pregnancy *(ref.)*								
No-birth-to-index-pregnancy	0.11	0.03	0.38	0.0004	0.10	0.03	0.32	0.0001
Years since abortion seeking	1.03	0.98	1.07	0.20	1.00	0.98	1.03	0.65
Constant	1.79	0.32	10.02	0.51	4.41	0.91	21.27	0.06

Panel B: Model-predicted probabilities of a subsequent birth being undesired (before conception).								
	Unweighted model				Weighted model			
	Predicted probability	Lower CI	Upper CI	p-value	Predicted probability	Lower CI	Upper CI	p-value
Gave-birth-to-index-pregnancy	77.9%	59.2%	96.7%	–	84.5%	69.0%	99.9%	–
No-birth-to-index-pregnancy	36.5%	30.5%	42.5%	–	35.1%	26.4%	43.8%	–
Difference, percentage points	41.4	23.1	59.7	<0.0001	49.4	33.8	65.0	<0.0001

**Notes:** Models included study group, years since abortion seeking as a continuous variable, and a random intercept for each mother to account for multiple births per individual, with cluster-robust standard errors at the facility level. The weighted model included propensity-score weights to balance baseline characteristics across study groups. A birth was classified as undesired if the mother’s most recent pre-pregnancy DAP score was >2.5, indicating a strong desire to avoid pregnancy. Among the 481 subsequent (post-index) births, 458 occurred to 420 women in the *no-birth-to-index-pregnancy* group and 23 occurred to 22 women in the *gave-birth-to-index-pregnancy* group. Pre-pregnancy DAP scores were available for 471 births; ten births (nine in the *no-birth from index pregnancy* group and one in the *gave-birth to index pregnancy* group) lacked pre-pregnancy DAP scores and were excluded. DAP = Desire to Avoid Pregnancy scale; AOR = adjusted odds ratio; CI = confidence interval.

In a sensitivity analysis, 43.2% of subsequent births in the *no-birth-to-index-pregnancy* group were preceded by an openness to pregnancy (DAP ≤ 1.5) compared with 13.3% in the *gave-birth-to-index-pregnancy* group, a difference of 30.0 percentage points (95% CI: 14.7–45.2; p = 0.0001; Supplementary Table S6).

Across all analyses, results were substantively unchanged after excluding women whose index pregnancy ended in miscarriage (Supplementary Tables S7–S12).

## Discussion

In this five-year prospective cohort study of Nepali women seeking abortions, whether or not women were able to avoid an undesired birth was associated with distinct reproductive trajectories over the subsequent years. Compared with women who were denied abortion access and gave birth, those who received abortions were more open to potential childbearing in the following years, more likely to become pregnant and to give birth after returning to fecundability, and more likely to have subsequent births that reflected their fertility preferences. Findings suggest that access to abortion can support reproductive autonomy both at the time of abortion seeking and in the years that follow, enabling some individuals to have children at times that better align with their evolving intentions and circumstances.

At the time of abortion seeking, lifetime fertility desires were similar between study groups: more than one third of women wanted to have a child in the future. For some, abortion may therefore have been a means of delaying childbearing until circumstances or timing were more favorable. In the four years after abortion seeking, women who received abortions expressed greater openness to pregnancy than those who were denied and gave birth. This pattern may reflect that women denied abortions who gave birth had already achieved a larger share of their desired lifetime fertility through the denied pregnancy, reducing their openness to another pregnancy in the near term. For these women, the experience of having had a child, particularly from a pregnancy they sought to end, may have also contributed to their stronger desire to avoid pregnancy in subsequent years.

Importantly, the implications of abortion access for women’s reproductive autonomy appear to extend beyond the index pregnancy. While relatively few subsequent births occurred among women who gave birth following abortion denial, subsequent births among women who received an abortion were substantially less likely to be undesired (before conception). Ideally, alignment between fertility preferences and childbearing would have also been assessed by comparing women’s DAP scores prior to the index birth with those for subsequent births. Such a comparison is not possible because participants were already pregnant at baseline. However, low LMUP scores at baseline indicate that index pregnancies occurred in the context of low planning or intendedness; participants’ abortion-seeking also signals that they did not want to carry those pregnancies to term. Based on these assumptions, our findings suggest that access to a desired abortion not only prevented an undesired index birth but also increased the likelihood that subsequent births occurred in alignment with women’s fertility preferences. Conversely, for women who were denied abortions and gave birth, the denial may have led to a double constraint on their reproductive autonomy: they were forced to carry the undesired index pregnancy to term and their subsequent births were more likely to be undesired prior to conception.

Prior research shows that the consequences of having an undesired birth can extend across multiple domains of women’s lives, including their physical and mental health, economic security, intimate partnerships, and life plans.^30^, ^31^, ^32^, ^33^, ^34^ Other analyses from the *Nepal Turnaway Study* found that carrying an unwanted pregnancy to term was associated with greater economic hardship over the following five years,^35^ whereas avoiding an unwanted birth was associated with greater life satisfaction,^36^ reduced intimate partner violence,^37^ and greater decision-making power within the household.^38^ These findings highlight the need to ensure that people denied abortion care receive continued support after the index birth, including postpartum contraceptive counseling, access to the full range of contraceptive methods, and safe abortion services if they later experience another undesired pregnancy.

Additionally, we found that women who received abortion care experienced a shorter time to subsequent pregnancy and birth after a return to fecundability than those who gave birth to the index pregnancy. Interpretation of these findings is limited, however, because our study was not designed to assess biological fecundity, infertility, or women’s ability to achieve a desired pregnancy. Future research incorporating data on proactive pregnancy intentions and attempts to conceive could help to distinguish biological fecundity from reproductive preferences and behaviors, and to assess whether women were able to realize desires to give birth. Although a prior review of the evidence found no causal relationship between safe abortion and infertility,^39^ this distinction remains important in contexts where misinformation persists, including Nepal.^40^

Furthermore, despite earlier subsequent childbearing among women who received abortions, cumulative births by year five remained higher among women who were denied and gave birth. Longer follow-up is needed to assess whether this difference persists to completed family size by the end of women’s reproductive years.

We acknowledge several limitations of this study. First, randomization of women to receipt of abortion is not feasible. While the study included only women seeking abortion, those who received abortions may have differed systematically from those denied in ways that affect fertility preferences or outcomes. Although propensity scores balanced measured confounders, unmeasured factors may still have influenced both abortion access and subsequent fertility trajectories. Second, loss to follow-up or administrative censoring may have limited power to detect differences in later outcomes. However, attrition was not differential by study group, and administrative censoring was related to the timing of recruitment and largely accounted for through propensity scores incorporating baseline characteristics. Third, the DAP scale has not undergone psychometric testing in Nepal; nevertheless, our tests showed high internal consistency. Fourth, analyses of subsequent births among women who were denied abortion and gave birth were based on a small number of births, limiting the precision of those estimates.

However, this study has notable strengths. The comparison group of women who were denied abortions and gave birth provides a unique counterfactual for assessing how abortion access shapes subsequent reproductive trajectories. The longitudinal design enabled us to explore how abortion access was associated with both the number of children women had and the extent to which subsequent births aligned with their reproductive preferences in the years after abortion seeking. Finally, prospective measurement of pregnancy preferences captured how fertility preferences evolved over time, addressing some of the challenges involved in research on fertility preferences.

Our results show that abortion denial, which persists even in this enabling policy context, can shape women’s reproductive trajectories for years after abortion seeking. Ultimately, these findings underscore the importance of ensuring equitable access to safe, legal abortion as part of comprehensive reproductive healthcare to support individuals in fulfilling their reproductive desires across the life course.

## Contributors

CM contributed to literature search, figures, study design, data analysis, data interpretation, and writing (original draft and editing). DGF contributed to study design, data collection, data analysis, data interpretation, writing (review and editing), and was responsible for conceptualization and funding acquisition. RM contributed to data analysis, data interpretation, and writing (review and editing). MP contributed to study design, data collection, data interpretation, and writing (review and editing). CHR contributed to data analysis, data interpretation, writing (review and editing), and supervision.

## Data sharing statement

Proposals should be directed to diana.greenefoster@ucsf.edu; to gain access, data requestors will need to sign a data access agreement. Data are available for 5 years at a third-party website: https://dataverse.harvard.edu/dataset.xhtml?persistentId=doi:10.7910/DVN/HMOWCA.

## Declaration of interests

The authors have declared that no competing interests exist.

## References

[1] Ministry of Health and Population/Nepal, New ERA, ICF (2023). https://www.dhsprogram.com/pubs/pdf/FR379/FR379.pdf.

[2] United Nations Population Fund (UNFPA) (1994).

[3] Gipson J.D., Koenig M.A., Hindin M.J. (2008). The effects of unintended pregnancy on infant, child, and parental health: a review of the literature. Stud Fam Plann.

[4] Hall J.A., Benton L., Copas A., Stephenson J. (2017). Pregnancy intention and pregnancy outcome: systematic review and meta-analysis. Matern Child Health J.

[5] Tsui A.O., McDonald-Mosley R., Burke A.E. (2010). Family planning and the burden of unintended pregnancies. Epidemiol Rev.

[6] Shah P.S., Taiba B., Ohlsson A. (2009). Intention to become pregnant and low birth weight and preterm birth: a systematic review. Matern Child Health J.

[7] Biggs M.A., Gould H., Foster D.G. (2013). Understanding why women seek abortions in the US. BMC Womens Health.

[8] Jones R.K., Foster D.G., Biggs M.A. (2021). Fertility intentions and recent births among US abortion patients. Contraception.

[9] Nepal Ministry of Health (2002).

[10] Nepal Law Commission, Government of Nepal (2018).

[11] Samandari G., Wolf M., Basnett I., Hyman A., Andersen K. (2012). Implementation of legal abortion in Nepal: a model for rapid scale-up of high-quality care. Reprod Health.

[12] Andersen K.L., Basnett I., Shrestha D.R., Shrestha M.K., Shah M., Aryal S. (2016). Expansion of safe abortion services in Nepal through auxiliary nurse-midwife provision of medical abortion, 2011-2013. J Midwifery Womens Health.

[13] Wu W.-J., Maru S., Regmi K., Basnett I. (2017). Abortion care in Nepal, 15 years after legalization: gaps in access, equity, and quality. Health Hum Rights.

[14] Puri S., Sundaram H., Tamang C. (2016). Abortion incidence and unintended pregnancy in Nepal. Int Perspect Sex Reprod Health.

[15] Rocca C., Puri M., Dulal B. (2013). Unsafe abortion after legalisation in Nepal: a cross-sectional study of women presenting to hospitals. BJOG.

[16] Puri M.C., Raifman S., Khanal B., Maharjan D.C., Foster D.G. (2018). Providers’ perspectives on denial of abortion care in Nepal: a cross sectional study. Reprod Health.

[17] Thapa S., Sharma S., Khatiwada N. (2014). Women’s knowledge of abortion law and availability of services in Nepal. J Biosoc Sci.

[18] Puri M., Vohra D., Gerdts C., Foster D.G. (2015). “I need to terminate this pregnancy even if it will take my life”: a qualitative study of the effect of being denied legal abortion on women’s lives in Nepal. BMC Womens Health.

[19] Puri M.C., Raifman S., Daniel S. (2023). Denial of legal abortion in Nepal. PLoS One.

[20] Bhrolcháin M.N., Beaujouan É. (2019). Springer series on demographic methods and population analysis.

[21] Müller M.W., Hamory J., Johnson-Hanks J., Miguel E. (2022). The illusion of stable fertility preferences. Popul Stud (NY).

[22] Iacovou M., Tavares L.P. (2011). Yearning, learning, and conceding: reasons men and women change their childbearing intentions. Popul Dev Rev.

[23] Hayford S.R. (2009). The evolution of fertility expectations over the life course. Demography.

[24] Conde-Agudelo A., Rosas-Bermúdez A., Kafury-Goeta A.C. (2006). Birth spacing and risk of adverse perinatal outcomes: a meta-analysis. JAMA.

[25] Upadhyay U.D., Aztlan-James E.A., Rocca C.H., Foster D.G. (2019). Intended pregnancy after receiving vs. being denied a wanted abortion. Contraception.

[26] Murro R., Raifman S., Boscardin J. (2026). Cohort profile: the Nepal turnaway study, A prospective cohort of abortion-seekers. medRxiv.

[27] Rocca C.H., Ralph L.J., Wilson M., Gould H., Foster D.G. (2019). Psychometric evaluation of an instrument to measure prospective pregnancy preferences. Med Care.

[28] Rocca C.H., Bullington B.W., Kavanaugh M., Stulberg D., Borrero S., Muñoz I. (2025).

[29] Lambert P.C., Royston P. (2009). Further development of flexible parametric models for survival analysis. STATA J.

[30] Biggs M.A., Upadhyay U.D., McCulloch C.E., Foster D.G. (2017). Women’s mental health and well-being 5 years after receiving or being denied an abortion: a prospective, longitudinal cohort study. JAMA Psychiatry.

[31] Ralph L.J., Schwarz E.B., Grossman D., Foster D.G. (2019). Self-reported physical health of women who did and did not terminate pregnancy after seeking abortion services: a cohort study. Ann Intern Med.

[32] Roberts S.C.M., Biggs M.A., Chibber K.S., Gould H., Rocca C.H., Foster D.G. (2014). Risk of violence from the man involved in the pregnancy after receiving or being denied an abortion. BMC Med.

[33] Foster D.G., Biggs M.A., Ralph L., Gerdts C., Roberts S., Glymour M.M. (2018). Socioeconomic outcomes of women who receive and women who are denied wanted abortions in the United States. Am J Public Health.

[34] Upadhyay U.D., Foster D.G., Gould H., Biggs M.A. (2022). Intimate relationships after receiving versus being denied an abortion: a 5-year prospective study in the United States. Perspect Sex Reprod Health.

[35] Puri M.C., Murro R., Karki S., Maharjan D.C., Diamond-Smith N.G., Foster D.G. (2026).

[36] Biggs M.A., Murro R., Puri M.C., Magar A., Foster D.G. (2026).

[37] Murro R., Diamond-Smith N.G., Puri M.C., Magar A., Foster D.G. (2026).

[38] Diamond-Smith N.G., Foster D.G., Puri M.C., Murro R., Magar A.A., Biggs M.A. (2026).

[39] Hogue C.J.R. (1986). Impact of abortion on subsequent fecundity. Clin Obstet Gynaecol.

[40] Ghimire R., Sharma S., Singh A., Bhusal R., Tamang L. (2025). Barriers to accessibility and availability of safe abortion services among young women in Nepal: a mixed-methods study. BMC Public Health.

