# Supplementary material for: Access to abortion and subsequent fertility preferences and reproductive outcomes in Nepal (2019–2024): a prospective cohort study
Source: Lancet Reg Health Southeast Asia. 2026 Sep 3;52:100832. doi: 10.1016/j.lansea.2026.100832 (PMC13711062; doi:10.1016/j.lansea.2026.100832)
Supplement: Supplementary file 1 [file mmc1.docx]

| **Supplementary Table 1.** Model-predicted probabilities of pregnancy preferences and pregnancy status after abortion seeking, by study group. | | | | | | | | |  |
| --- | --- | --- | --- | --- | --- | --- | --- | --- | --- |
|  |  | **No-birth-to-index-pregnancy group** | | **Gave-birth-to-index-pregnancy group** | |  |  |  | |
| **Pregnancy preferences and pregnancy status** | **Years since abortion seeking** | Marginal predicted probability | SE | Marginal predicted probability | SE | **Risk difference** (Gave-birth-to-index-pregnancy vs. No-birth-to-index-pregnancy) | SE | p-value | |
| ***Strong desire to avoid pregnancy***  (DAP >2·5 to 4) | 1 | 0·674 | 0·020 | 0·949 | 0·015 | 0·275 | 0·022 | <0·0001 | |
|  | 2 | 0·709 | 0·020 | 0·933 | 0·013 | 0·223 | 0·021 | <0·0001 | |
|  | 3 | 0·742 | 0·023 | 0·910 | 0·014 | 0·169 | 0·023 | <0·0001 | |
|  | 4 | 0·770 | 0·027 | 0·880 | 0·019 | 0·110 | 0·030 | 0·002 | |
|  | 5 | 0·796 | 0·030 | 0·837 | 0·031 | 0·042 | 0·040 | 0·30 | |
| ***Moderate desire to avoid pregnancy***  (DAP >1·5 to 2·5) | 1 | 0·103 | 0·013 | 0·033 | 0·013 | -0·070 | 0·013 | <0·0001 | |
|  | 2 | 0·094 | 0·010 | 0·040 | 0·011 | -0·054 | 0·011 | <0·0001 | |
|  | 3 | 0·085 | 0·009 | 0·048 | 0·009 | -0·037 | 0·010 | 0·002 | |
|  | 4 | 0·077 | 0·009 | 0·057 | 0·013 | -0·020 | 0·013 | 0·14 | |
|  | 5 | 0·069 | 0·010 | 0·067 | 0·023 | -0·002 | 0·022 | 0·92 | |
| ***Open to pregnancy***  (DAP ≤ 1·5) | 1 | 0·136 | 0·023 | 0·008 | 0·004 | -0·128 | 0·021 | <0·0001 | |
|  | 2 | 0·128 | 0·021 | 0·015 | 0·005 | -0·113 | 0·018 | <0·0001 | |
|  | 3 | 0·121 | 0·021 | 0·028 | 0·006 | -0·093 | 0·019 | <0·0001 | |
|  | 4 | 0·113 | 0·023 | 0·047 | 0·009 | -0·066 | 0·021 | 0·002 | |
|  | 5 | 0·105 | 0·026 | 0·078 | 0·017 | -0·026 | 0·024 | 0·28 | |
| ***Currently pregnant***  (no DAP scale score) | 1 | 0·088 | 0·009 | 0·011 | 0·005 | -0·077 | 0·012 | <0·0001 | |
|  | 2 | 0·068 | 0·005 | 0·012 | 0·003 | -0·056 | 0·007 | <0·0001 | |
|  | 3 | 0·053 | 0·004 | 0·014 | 0·003 | -0·039 | 0·004 | <0·0001 | |
|  | 4 | 0·040 | 0·004 | 0·016 | 0·005 | -0·024 | 0·004 | <0·0001 | |
|  | 5 | 0·030 | 0·004 | 0·017 | 0·008 | -0·013 | 0·007 | 0·08 | |
| **Notes:** Values represent marginal predicted probabilities from a mixed-effects multinomial logistic regression model with a categorical outcome: (1) strong desire to avoid pregnancy, (2) moderate desire to avoid pregnancy, (3) open to pregnancy, or (4) currently pregnant. The model included study group, time since abortion seeking (in years), and their interaction, with individual-level random intercepts to account for within-person correlation across repeated observations. Cluster-robust standard errors at the facility level and propensity score weights were applied. Model-predicted probabilities and risk differences between study groups were obtained using the *margins* command in Stata. DAP = Desire to Avoid Pregnancy scale; SE = standard error. | | | | | | | | | |

| **Supplementary Table 2.** Adjusted relative risk ratios (ARRRs) from a mixed-effects multinomial logistic regression model of pregnancy preferences and pregnancy status over time, by study group. | | | | |
| --- | --- | --- | --- | --- |
|  | **ARRR** | **Lower CI** | **Upper CI** | **p-value** |
| **Currently pregnant (no DAP scale score)** |  |  |  |  |
| Study group: Gave-birth-to-index-pregnancy group (ref.) |  |  |  |  |
| No-birth-to-index-pregnancy group | 21·21 | 4·95 | 90·88 | <0·0001 |
| Years since abortion seeking | 1·14 | 0·75 | 1·74 | 0·53 |
| Gave-birth-to-index-pregnancy group × Years since abortion seeking |  |  |  |  |
| No-birth-to-index-pregnancy group × Years since abortion seeking | 0·63 | 0·40 | 0·98 | 0·042 |
| Constant | 0·005 | 0·001 | 0·02 | <0·0001 |
| **Open to pregnancy (DAP ≤ 1·5)** |  |  |  |  |
| Study group: Gave-birth-to-index-pregnancy group (ref.) |  |  |  |  |
| No-birth-to-index-pregnancy group | 308·83 | 94·89 | 1005·16 | <0·0001 |
| Years since abortion seeking | 2·43 | 1·78 | 3·31 | <0·0001 |
| Gave-birth-to-index-pregnancy group × Years since abortion seeking |  |  |  |  |
| No-birth-to-index-pregnancy group × Years since abortion seeking | 0·35 | 0·26 | 0·47 | <0·0001 |
| Constant | 0·0001 | 0·00003 | 0·0005 | <0·0001 |
| **Moderate desire to avoid pregnancy (DAP >1·5 to 2·5)** |  |  |  |  |
| Study group: Gave-birth-to-index-pregnancy group (ref.) |  |  |  |  |
| No-birth-to-index-pregnancy group | 9·41 | 2·41 | 36·81 | 0·001 |
| Years since abortion seeking | 1·29 | 0·84 | 1·97 | 0·24 |
| Gave-birth-to-index-pregnancy group × Years since abortion seeking |  |  |  |  |
| No-birth-to-index-pregnancy group × Years since abortion seeking | 0·65 | 0·44 | 0·96 | 0·033 |
| Constant | 0·007 | 0·001 | 0·05 | <0·0001 |
| **Notes**: Changes in pregnancy preferences and pregnancy status from one through five years after abortion seeking were examined using a mixed-effects multinomial logistic regression model. The categorical outcome was defined as: (1) strong desire to avoid pregnancy, (2) moderate desire to avoid pregnancy, (3) open to pregnancy, or (4) currently pregnant. Strong desire to avoid pregnancy (DAP score >2·5 to 4) was the referent outcome. The model included study group, time since abortion seeking (in years), and their interaction, with individual-level random intercepts to account for within-person correlation across repeated observations. Propensity score weights were applied and standard errors were clustered at the facility level. DAP = Desire to Avoid Pregnancy scale; CI = confidence interval. | | | | |

| **Supplementary Table 3.** Model-predicted cumulative incidence, survival probability, hazard rate, and hazard ratio for subsequent pregnancy and birth, by years since entry into the risk period. | | | | | | | | | | | | | |
| --- | --- | --- | --- | --- | --- | --- | --- | --- | --- | --- | --- | --- | --- |
| **Outcome: Subsequent pregnancy** | | **Survival probability** | | | **Cumulative incidence** | | | **Hazard rate** | | | **Hazard ratio** | | |
| **Years since entry into the risk period** | **Study group** | % | Lower CI | Upper CI | % | Lower CI | Upper CI | Rate | Lower CI | Upper CI | Ratio | Lower CI | Upper CI |
| 1 | No-birth-to-index-pregnancy | 83·8 | 81·3 | 86·4 | 16·2 | 13·6 | 18·7 | 0·15 | 0·12 | 0·20 | 4·68 | 2·69 | 8·13 |
|  | Gave-birth-to-index-pregnancy | 96·0 | 93·9 | 98·1 | 4·0 | 1·9 | 6·1 | 0·03 | 0·02 | 0·06 |  |  |  |
| 2 | No-birth-to-index-pregnancy | 72·0 | 69·0 | 75·1 | 28·0 | 24·9 | 31·0 | 0·16 | 0·13 | 0·20 | 2·68 | 1·79 | 4·02 |
|  | Gave-birth-to-index-pregnancy | 91·7 | 88·5 | 95·0 | 8·3 | 5·0 | 11·5 | 0·06 | 0·04 | 0·09 |  |  |  |
| 3 | No-birth-to-index-pregnancy | 63·0 | 59·6 | 66·6 | 37·0 | 33·4 | 40·4 | 0·10 | 0·08 | 0·12 | 1·74 | 1·08 | 2·80 |
|  | Gave-birth-to-index-pregnancy | 86·3 | 82·3 | 90·5 | 13·7 | 9·5 | 17·7 | 0·06 | 0·04 | 0·09 |  |  |  |
| 4 | No-birth-to-index-pregnancy | 58·2 | 54·7 | 61·9 | 41·8 | 38·1 | 45·3 | 0·06 | 0·05 | 0·09 | 1·21 | 0·55 | 2·66 |
|  | Gave-birth-to-index-pregnancy | 81·7 | 76·8 | 86·8 | 18·3 | 13·2 | 23·2 | 0·05 | 0·03 | 0·11 |  |  |  |
| **Outcome: Subsequent birth** | | **Survival probability** | | | **Cumulative incidence** | | | **Hazard rate** | | | **Hazard ratio** | | |
| **Years since entry into the risk period** | **Study group** | % | Lower CI | Upper CI | % | Lower CI | Upper CI | Rate | Lower CI | Upper CI | Ratio | Lower CI | Upper CI |
| 1 | No-birth-to-index-pregnancy | 97·8 | 97·1 | 98·5 | 2·2 | 1·5 | 2·9 | 0·12 | 0·09 | 0·16 | 5·08 | 2·10 | 12·27 |
|  | Gave-birth-to-index-pregnancy | 99·2 | 98·3 | 100·0 | 0·8 | 0·0 | 1·7 | 0·02 | 0·01 | 0·05 |  |  |  |
| 2 | No-birth-to-index-pregnancy | 84·8 | 82·3 | 87·4 | 15·2 | 12·6 | 17·7 | 0·09 | 0·06 | 0·13 | 5·53 | 2·34 | 13·07 |
|  | Gave-birth-to-index-pregnancy | 97·5 | 95·7 | 99·4 | 2·5 | 0·6 | 4·3 | 0·02 | 0·01 | 0·04 |  |  |  |
| 3 | No-birth-to-index-pregnancy | 75·9 | 72·9 | 79·0 | 24·1 | 21·0 | 27·1 | 0·12 | 0·09 | 0·16 | 2·70 | 1·56 | 4·66 |
|  | Gave-birth-to-index-pregnancy | 94·5 | 91·6 | 97·5 | 5·5 | 2·5 | 8·4 | 0·04 | 0·03 | 0·07 |  |  |  |
| 4 | No-birth-to-index-pregnancy | 69·2 | 65·8 | 72·7 | 30·8 | 27·3 | 34·2 | 0·07 | 0·05 | 0·09 | 1·34 | 0·35 | 5·07 |
|  | Gave-birth-to-index-pregnancy | 90·0 | 85·7 | 94·6 | 10·0 | 5·4 | 14·3 | 0·05 | 0·01 | 0·20 |  |  |  |
| **Notes:** Flexible parametric survival models were used to model time to the next (non-index) pregnancy and birth using the stpm2 module in Stata. Models included a time-varying study group effect to account for non-proportional hazards, as well as propensity score weights and cluster-robust standard errors at the facility level. For the *gave-birth-to-index-pregnancy* group, entry into the risk period was set to three months after the index birth to account for the postpartum period during which conception was unlikely. For the *no-birth-to-index-pregnancy* group, entry into the risk period was set to one month after abortion seeking. Follow-up was administratively censored at 1,888 days (~5·2 years) for the *no-birth-to-index-pregnancy* group and 1,500 days (~4·1 years) for the *gave-birth-to-index-pregnancy* group. To enable comparability across study groups, we present results up through four years since entry into the risk period, when both groups had sufficient follow-up data. Cumulative incidence was calculated as 1 minus the model-predicted survival probability. Hazard ratios compare the *no-birth-to-index-pregnancy* group with the *gave-birth-to-index-pregnancy* group. CI = confidence interval. | | | | | | | | | | | | | |

| **Supplementary Table 4.** Sensitivity analysis using six-month postpartum entry into the risk period for the *gave-birth-to-index-pregnancy* group. Model-predicted cumulative incidence, survival probability, hazard rate, and hazard ratio for subsequent pregnancy and birth, by years since entry into the risk period. | | | | | | | | | | | | | |
| --- | --- | --- | --- | --- | --- | --- | --- | --- | --- | --- | --- | --- | --- |
| **Outcome: Subsequent pregnancy** | | **Survival probability** | | | **Cumulative incidence** | | | **Hazard rate** | | | **Hazard ratio** | | |
| **Years since entry into the risk period** | **Study group** | % | Lower CI | Upper CI | % | Lower CI | Upper CI | Rate | Lower CI | Upper CI | Ratio | Lower CI | Upper CI |
| 1 | No-birth-to-index-pregnancy | 83·8 | 81·2 | 86·4 | 16·2 | 13·6 | 18·8 | 0·16 | 0·13 | 0·21 | 4·23 | 2·46 | 7·27 |
|  | Gave-birth-to-index-pregnancy | 97·0 | 95·4 | 98·6 | 3·0 | 1·4 | 4·6 | 0·04 | 0·02 | 0·07 |  |  |  |
| 2 | No-birth-to-index-pregnancy | 71·7 | 68·8 | 74·8 | 28·3 | 25·2 | 31·2 | 0·15 | 0·12 | 0·19 | 2·55 | 1·65 | 3·92 |
|  | Gave-birth-to-index-pregnancy | 92·3 | 89·1 | 95·5 | 7·7 | 4·5 | 10·9 | 0·06 | 0·04 | 0·09 |  |  |  |
| 3 | No-birth-to-index-pregnancy | 63·1 | 59·7 | 66·7 | 36·9 | 33·3 | 40·3 | 0·10 | 0·08 | 0·12 | 1·67 | 0·97 | 2·90 |
|  | Gave-birth-to-index-pregnancy | 86·8 | 82·8 | 90·9 | 13·2 | 9·1 | 17·2 | 0·06 | 0·04 | 0·10 |  |  |  |
| 4 | No-birth-to-index-pregnancy | 58·3 | 54·8 | 61·9 | 41·7 | 38·1 | 45·2 | 0·07 | 0·05 | 0·09 | 1·20 | 0·48 | 3·02 |
|  | Gave-birth-to-index-pregnancy | 82·0 | 76·7 | 87·6 | 18·0 | 12·4 | 23·3 | 0·05 | 0·02 | 0·13 |  |  |  |
| **Outcome: Subsequent birth** | | **Survival probability** | | | **Cumulative incidence** | | | **Hazard rate** | | | **Hazard ratio** | | |
| **Years since entry into the risk period** | **Study group** | % | Lower CI | Upper CI | % | Lower CI | Upper CI | Rate | Lower CI | Upper CI | Ratio | Lower CI | Upper CI |
| 1 | No-birth-to-index-pregnancy | 97·7 | 97·0 | 98·4 | 2·3 | 1·6 | 3·0 | 0·12 | 0·09 | 0·16 | 4·80 | 2·07 | 11·12 |
|  | Gave-birth-to-index-pregnancy | 98·6 | 97·4 | 99·8 | 1·4 | 0·2 | 2·6 | 0·03 | 0·01 | 0·05 |  |  |  |
| 2 | No-birth-to-index-pregnancy | 84·8 | 82·3 | 87·4 | 15·2 | 12·6 | 17·7 | 0·10 | 0·07 | 0·14 | 4·51 | 2·28 | 8·93 |
|  | Gave-birth-to-index-pregnancy | 96·8 | 94·7 | 99·0 | 3·2 | 1·0 | 5·3 | 0·02 | 0·01 | 0·04 |  |  |  |
| 3 | No-birth-to-index-pregnancy | 75·7 | 72·8 | 78·8 | 24·3 | 21·2 | 27·2 | 0·11 | 0·09 | 0·15 | 2·95 | 1·71 | 5·07 |
|  | Gave-birth-to-index-pregnancy | 93·6 | 90·6 | 96·7 | 6·4 | 3·3 | 9·4 | 0·04 | 0·02 | 0·07 |  |  |  |
| 4 | No-birth-to-index-pregnancy | 69·3 | 66·0 | 72·8 | 30·7 | 27·2 | 34·0 | 0·07 | 0·05 | 0·09 | 2·39 | 0·67 | 8·53 |
|  | Gave-birth-to-index-pregnancy | 90·5 | 86·6 | 94·6 | 9·5 | 5·4 | 13·4 | 0·03 | 0·01 | 0·10 |  |  |  |
| **Notes:** Flexible parametric survival models were used to model time to the next (non-index) pregnancy and birth using the stpm2 module in Stata. Models included a time-varying study group effect to account for non-proportional hazards, as well as propensity score weights and cluster-robust standard errors at the facility level. For the *gave-birth-to-index-pregnancy* group, entry into the risk period was set to six months after the index birth to account for the postpartum period during which conception was unlikely. For the *no-birth-to-index-pregnancy* group, entry into the risk period was set to one month after abortion seeking. Follow-up was administratively censored at 1,888 days (~5·2 years) for the *no-birth-to-index-pregnancy* group and 1,500 days (~4·1 years) for the *gave-birth-to-index-pregnancy* group. To enable comparability across study groups, we present results up through four years since entry into the risk period, when both groups had sufficient follow-up data. Cumulative incidence was calculated as 1 minus the model-predicted survival probability. Hazard ratios compare the *no-birth-to-index-pregnancy* group with the *gave-birth-to-index-pregnancy* group. CI = confidence interval. | | | | | | | | | | | | | |

| **Supplementary Table 5.** Incidence-rate ratios (IRRs) from a mixed-effects Poisson regression model estimating the number of children born over the five-year period, by study group. | | | | |
| --- | --- | --- | --- | --- |
|  | **IRR** | **Lower CI** | **Upper CI** | **p-value** |
| **Study group:** Gave-birth-to-index-pregnancy group (ref.) |  |  |  |  |
| No-birth-to-index-pregnancy group | 0·003 | 0·002 | 0·01 | <0·0001 |
| **Linear term (time)** |  |  |  |  |
| Years since abortion seeking | 0·53 | 0·08 | 3·31 | 0·50 |
| No-birth-to-index-pregnancy group × Years since abortion seeking | 36·53 | 5·76 | 231·84 | <0·0001 |
| **Quadratic term (time²)** |  |  |  |  |
| Years since abortion seeking² | 0·30 | 0·07 | 1·33 | 0·11 |
| No-birth-to-index-pregnancy group × Years since abortion seeking² | 1·28 | 0·29 | 5·72 | 0·75 |
| **Cubic term (time³)** |  |  |  |  |
| Years since abortion seeking³ | 1·26 | 1·01 | 1·56 | 0·039 |
| No-birth-to-index-pregnancy group × Years since abortion seeking³ | 0·87 | 0·70 | 1·08 | 0·20 |
| **Constant** | 2·52 | 1·66 | 3·80 | <0·0001 |
| **Notes:** The number of children born over the five-year period was modeled using mixed-effects Poisson regression, with study group, time since abortion seeking (in years), and their interaction as predictors. Time was modeled using cubic polynomial terms and their interactions with study group to allow for non-linear fertility trajectories. Person-time between interviews (in years) was included as an offset to estimate birth rates. The model included individual-level random intercepts, propensity score weights, and facility-level cluster-robust standard errors. The *no-birth-to-index-pregnancy* group included participants who received an abortion on the day that they were recruited or were no longer pregnant at the first six-week follow-up interview, because they had an abortion or miscarried after initially being denied the abortion (n=1467). A total of 458 post-index births occurred among 420 women in the *no-birth-to-index-pregnancy* group. The *gave-birth-to-index-pregnancy* group included participants who were denied the abortion at baseline and gave birth (n=275). The *gave-birth-to-index-pregnancy* group had 275 index births (one per participant) and 23 post-index births among 22 women. CI=confidence interval. | | | | |

| **Supplementary Table 6.** Mixed-effects logistic regression estimates and model-predicted probabilities that subsequent births were desired, meaning preceded by an openness to pregnancy, by study group. | | | | | | | | |
| --- | --- | --- | --- | --- | --- | --- | --- | --- |
| **Panel A:**  Adjusted odds that subsequent births were preceded by an openness to pregnancy. | | | | | | | | |
|  | **Unweighted model** | | | | **Weighted model** | | | |
| **Predictor** | AOR | Lower CI | Upper CI | p-value | AOR | Lower CI | Upper CI | p-value |
| Gave-birth-to-index-pregnancy group *(ref.)* | |  |  |  |  |  |  |  |
| No-birth-to-index-pregnancy group | 4·53 | 1·50 | 13·66 | 0·007 | 5·11 | 1·53 | 16·99 | 0·008 |
| Years since abortion seeking | 1·00 | 0·98 | 1·03 | 0·66 | 1·02 | 1·01 | 1·03 | 0·006 |
| Constant | 0·11 | 0·03 | 0·44 | 0·002 | 0·07 | 0·02 | 0·32 | 0·0005 |
| **Panel B:** Model-predicted probabilities of a subsequent birth being preceded by an openness to pregnancy. | | | | | | | | |
|  | **Unweighted model** | | | | **Weighted model** | | | |
|  | Predicted probability | Lower CI | Upper CI | p-value | Predicted probability | Lower CI | Upper CI | p-value |
| Gave-birth-to-index-pregnancy group | 17·9% | 12·7% | 34·6% |  | 13·3% | 0·65% | 27·2% |  |
| No-birth-to-index-pregnancy group | 40·7% | 30·9% | 50·6% |  | 43·2% | 32·4% | 54·0% |  |
| Difference, percentage points | 22·7 | 6·4 | 39·2 | 0·007 | 30·0 | 14·7 | 45·2 | 0·0001 |
| **Notes:** Models included study group, years since abortion seeking as a continuous variable, and a random intercept for each mother to account for multiple births per individual, with cluster-robust standard errors at the facility level. The weighted model included propensity-score weights to balance baseline characteristics across study groups. A birth was classified as desired if the mother’s most recent pre-pregnancy DAP score was ≤1·5, indicating an openness to pregnancy. Among 481 subsequent (post-index) births, 458 births occurred to 420 women in the *no-birth-to-index-pregnancy* group and 23 births to 22 women in the *gave-birth-to-index-pregnancy* group. Pre-pregnancy DAP scores were available for 471 births; ten births (nine in the *no-birth-to-index-pregnancy* group and one in the *gave-birth-to-index-pregnancy* group) lacked pre-pregnancy DAP scores and were excluded. DAP = Desire to Avoid Pregnancy scale; AOR = adjusted odds ratio; CI = confidence interval. | | | | | | | | |

# **Sensitivity analyses excluding women who miscarried the index pregnancy (n=41).**

| **Supplementary Table 7.** Sensitivity analysis excluding women who miscarried the index pregnancy: Adjusted relative risk ratios (ARRRs) from a mixed-effects multinomial logistic regression model of pregnancy preferences and pregnancy status over time, by study group. | | | | |
| --- | --- | --- | --- | --- |
|  | **ARRR** | **Lower CI** | **Upper CI** | **p**-**value** |
| **Currently pregnant (no DAP scale score)** |  |  |  |  |
| Study group: Gave-birth-to-index-pregnancy group (ref.) |  |  |  |  |
| Received-abortion group | 21·86 | 5·07 | 94·26 | <0·0001 |
| Years since abortion seeking | 1·14 | 0·75 | 1·73 | 0·53 |
| Gave-birth-to-index-pregnancy group × Years since abortion seeking |  |  |  |  |
| Received-abortion group × Years since abortion seeking | 0·62 | 0·39 | 0·97 | 0·038 |
| Constant | 0·006 | 0·001 | 0·02 | <0·0001 |
|  |  |  |  |  |
| **Open to pregnancy (DAP ≤ 1·5)** |  |  |  |  |
| Study group: Gave-birth-to-index-pregnancy group (ref.) |  |  |  |  |
| Received-abortion group | 302·09 | 94·33 | 967·42 | <0·0001 |
| Years since abortion seeking | 2·42 | 1·78 | 3·29 | <0·0001 |
| Gave-birth-to-index-pregnancy group × Years since abortion seeking |  |  |  |  |
| Received-abortion group × Years since abortion seeking | 0·36 | 0·27 | 0·48 | <0·0001 |
| Constant | 0·0001 | 0·00003 | 0·0006 | <0·0001 |
|  |  |  |  |  |
| **Moderate desire to avoid pregnancy (DAP >1·5 to 2·5)** |  |  |  |  |
| Study group: Gave-birth-to-index-pregnancy group (ref.) |  |  |  |  |
| Received-abortion group | 9·41 | 2·40 | 36·82 | 0·001 |
| Years since abortion seeking | 1·29 | 0·84 | 1·97 | 0·24 |
| Gave-birth-to-index-pregnancy group × Years since abortion seeking |  |  |  |  |
| Received-abortion group × Years since abortion seeking | 0·66 | 0·44 | 0·98 | 0·037 |
| Constant | 0·007 | 0·001 | 0·05 | <0·0001 |
| **Notes:** Changes in pregnancy preferences and pregnancy status from one through five years after abortion seeking were examined using a mixed-effects multinomial logistic regression model. The categorical outcome was defined as: (1) strong desire to avoid pregnancy, (2) moderate desire to avoid pregnancy, (3) open to pregnancy, or (4) currently pregnant. Strong desire to avoid pregnancy (DAP score >2·5 to 4) was the referent outcome. The model included study group, time since abortion seeking (in years), and their interaction, with individual-level random intercepts to account for within-person correlation across repeated observations. Propensity score weights were applied and standard errors were clustered at the facility level. DAP = Desire to Avoid Pregnancy scale; CI = confidence interval. | | | | |

| **Supplementary Table 8.** Sensitivity analysis excluding women who miscarried the index pregnancy: Model-predicted probabilities of pregnancy preferences and pregnancy status after abortion seeking, by study group. | | | | | | | | |
| --- | --- | --- | --- | --- | --- | --- | --- | --- |
|  |  | **Received-abortion group** | | **Gave-birth-to-index-pregnancy group** | |  |  |  |
|  | **Years since abortion seeking** | Marginal predicted probability | SE | Marginal predicted probability | SE | **Risk difference** (Gave-birth-to-index-pregnancy vs. Received- abortion) | SE | p-value |
|  |  |  |  |  |  |  |  |  |
| **Pregnancy preferences and pregnancy status** |  |  |  |  |  |  |  |  |
| ***Strong desire to avoid pregnancy***  (DAP >2·5 to 4) | 1 | 0·671 | 0·017 | 0·949 | 0·015 | 0·278 | 0·021 | <0·0001 |
|  | 2 | 0·707 | 0·018 | 0·933 | 0·013 | 0·226 | 0·019 | <0·0001 |
|  | 3 | 0·739 | 0·021 | 0·911 | 0·014 | 0·172 | 0·022 | <0·0001 |
|  | 4 | 0·767 | 0·026 | 0·881 | 0·020 | 0·114 | 0·029 | <0·0001 |
|  | 5 | 0·792 | 0·030 | 0·838 | 0·031 | 0·047 | 0·040 | 0·24 |
| ***Moderate desire to avoid pregnancy***  (DAP >1·5 to 2·5) | 1 | 0·103 | 0·013 | 0·033 | 0·013 | -0·071 | 0·013 | <0·0001 |
|  | 2 | 0·095 | 0·010 | 0·040 | 0·011 | -0·055 | 0·011 | <0·0001 |
|  | 3 | 0·087 | 0·009 | 0·048 | 0·009 | -0·039 | 0·010 | <0·0001 |
|  | 4 | 0·079 | 0·009 | 0·057 | 0·013 | -0·022 | 0·014 | 0·11 |
|  | 5 | 0·072 | 0·010 | 0·067 | 0·023 | -0·005 | 0·022 | 0·83 |
| ***Open to pregnancy***  (DAP ≤ 1·5) | 1 | 0·136 | 0·022 | 0·007 | 0·003 | -0·128 | 0·020 | <0·0001 |
|  | 2 | 0·129 | 0·020 | 0·015 | 0·005 | -0·114 | 0·018 | <0·0001 |
|  | 3 | 0·122 | 0·020 | 0·027 | 0·006 | -0·094 | 0·018 | <0·0001 |
|  | 4 | 0·115 | 0·023 | 0·047 | 0·009 | -0·068 | 0·020 | 0·001 |
|  | 5 | 0·107 | 0·026 | 0·079 | 0·017 | -0·029 | 0·024 | 0·24 |
| ***Currently pregnant***  (no DAP scale score) | 1 | 0·090 | 0·010 | 0·011 | 0·005 | -0·079 | 0·013 | <0·0001 |
|  | 2 | 0·069 | 0·006 | 0·012 | 0·003 | -0·057 | 0·008 | <0·0001 |
|  | 3 | 0·052 | 0·004 | 0·013 | 0·003 | -0·039 | 0·004 | <0·0001 |
|  | 4 | 0·039 | 0·004 | 0·015 | 0·005 | -0·025 | 0·004 | <0·0001 |
|  | 5 | 0·029 | 0·004 | 0·016 | 0·008 | -0·013 | 0·008 | 0·08 |
| **Notes:** Values represent marginal predicted probabilities from a mixed-effects multinomial logistic regression model with a categorical outcome: (1) strong desire to avoid pregnancy, (2) moderate desire to avoid pregnancy, (3) open to pregnancy, or (4) currently pregnant. The model included study group, time since abortion seeking (in years), and their interaction, with individual-level random intercepts to account for within-person correlation across repeated observations. Cluster-robust standard errors at the facility level and propensity score weights were applied. Model-predicted probabilities and risk differences between study groups were obtained using the margins command in Stata. DAP = Desire to Avoid Pregnancy scale; SE = standard error. | | | | | | | | |

| **Supplementary Table 9.** Sensitivity analysis excluding women who miscarried the index pregnancy: Model-predicted cumulative incidence, survival probability, hazard rate, and hazard ratio for subsequent pregnancy and birth, by years since entry into the risk period. | | | | | | | | | | | | | |
| --- | --- | --- | --- | --- | --- | --- | --- | --- | --- | --- | --- | --- | --- |
| **Outcome: Subsequent pregnancy** | | **Survival probability** | | | **Cumulative incidence** | | | **Hazard rate** | | | **Hazard ratio** | | |
| **Years since entry into the risk period** | **Study group** | % | Lower CI | Upper CI | % | Lower CI | Upper CI | Rate | Lower CI | Upper CI | Ratio | Lower CI | Upper CI |
| 1 | Received-abortion | 83·7 | 81·2 | 86·4 | 16·2 | 13·6 | 18·8 | 0·15 | 0·12 | 0·20 | 4·69 | 2·70 | 8·15 |
|  | Gave-birth-to-index-pregnancy | 96·0 | 93·9 | 98·1 | 4·0 | 1·9 | 6·1 | 0·03 | 0·02 | 0·06 |  |  |  |
| 2 | Received-abortion | 71·9 | 68·9 | 75·0 | 28·1 | 25·0 | 31·1 | 0·16 | 0·13 | 0·20 | 2·69 | 1·79 | 4·03 |
|  | Gave-birth-to-index-pregnancy | 91·7 | 88·5 | 95·0 | 8·3 | 5·0 | 11·5 | 0·06 | 0·04 | 0·09 |  |  |  |
| 3 | Received-abortion | 62·9 | 59·5 | 66·5 | 37·1 | 33·5 | 40·5 | 0·10 | 0·08 | 0·12 | 1·75 | 1·09 | 2·81 |
|  | Gave-birth-to-index-pregnancy | 86·3 | 82·3 | 90·5 | 13·7 | 9·5 | 17·7 | 0·06 | 0·04 | 0·09 |  |  |  |
| 4 | Received-abortion | 58·0 | 54·6 | 61·7 | 41·9 | 38·2 | 45·4 | 0·06 | 0·05 | 0·09 | 1·22 | 0·55 | 2·66 |
|  | Gave-birth-to-index-pregnancy | 81·7 | 76·8 | 86·8 | 18·3 | 13·2 | 23·2 | 0·05 | 0·03 | 0·11 |  |  |  |
| **Outcome: Subsequent birth** | | **Survival probability** | | | **Cumulative incidence** | | | **Hazard rate** | | | **Hazard ratio** | | |
| **Years since entry into the risk period** | **Study group** | % | Lower CI | Upper CI | % | Lower CI | Upper CI | Rate | Lower CI | Upper CI | Ratio | Lower CI | Upper CI |
| 1 | Received-abortion | 97·8 | 97·1 | 98·5 | 2·2 | 1·5 | 2·9 | 0·12 | 0·09 | 0·16 | 5·09 | 2·11 | 12·30 |
|  | Gave-birth-to-index-pregnancy | 99·2 | 98·3 | 100·0 | 0·8 | 0·0 | 1·7 | 0·02 | 0·01 | 0·05 |  |  |  |
| 2 | Received-abortion | 84·8 | 82·3 | 87·4 | 15·2 | 12·6 | 17·7 | 0·09 | 0·06 | 0·13 | 5·54 | 2·34 | 13·12 |
|  | Gave-birth-to-index-pregnancy | 97·5 | 95·7 | 99·4 | 2·5 | 0·6 | 4·3 | 0·02 | 0·01 | 0·04 |  |  |  |
| 3 | Received-abortion | 75·8 | 72·9 | 78·9 | 24·2 | 21·1 | 27·1 | 0·12 | 0·09 | 0·16 | 2·71 | 1·57 | 4·68 |
|  | Gave-birth-to-index-pregnancy | 94·5 | 91·6 | 97·5 | 5·5 | 2·5 | 8·4 | 0·04 | 0·03 | 0·07 |  |  |  |
| 4 | Received-abortion | 69·1 | 65·7 | 72·6 | 30·9 | 27·4 | 34·3 | 0·07 | 0·05 | 0·10 | 1·34 | 0·35 | 5·10 |
|  | Gave-birth-to-index-pregnancy | 90·0 | 85·7 | 94·6 | 10·0 | 5·4 | 14·3 | 0·05 | 0·01 | 0·20 |  |  |  |
| **Notes:** Flexible parametric survival models were used to model time to the next (non-index) pregnancy and birth using the stpm2 module in Stata. Models included a time-varying study group effect to account for non-proportional hazards, as well as propensity score weights and cluster-robust standard errors at the facility level. For the *gave-birth-to-index-pregnancy* group, entry into the risk period was set to three months after the index birth to account for the postpartum period during which conception was unlikely. For the *received-abortion* group, entry into the risk period was set to one month after abortion seeking. Follow-up was administratively censored at 1,888 days (~5·2 years) for the *received-abortion* group and 1,500 days (~4·1 years) for the *gave-birth-to-index-pregnancy* group. To enable comparability across study groups, we present results up through four years since entry into the risk period, when both groups had sufficient follow-up data. Cumulative incidence was calculated as 1 minus the model-predicted survival probability. Hazard ratios compare the *received-abortion* group with the *gave-birth-to-index-pregnancy* group. | | | | | | | | | | | | | |

| **Supplementary Table 10.** Sensitivity analysis excluding women who miscarried the index pregnancy: Incidence-rate ratios (IRRs) from a mixed-effects Poisson regression model estimating the number of children born over the five-year period, by study group. | | | | |  |
| --- | --- | --- | --- | --- | --- |
|  | **IRR** | **Lower CI** | **Upper CI** | **p-value** |  |
|  |  |  |  |  |  |
| **Study group:** Gave-birth-to-index-pregnancy group (ref.) |  |  |  |  |  |
| Received-abortion group | 0·003 | 0·002 | 0·01 | <0·0001 |  |
| **Linear term (time)** |  |  |  |  |  |
| Years since abortion seeking | 0·53 | 0·08 | 3·31 | 0·50 |  |
| Received-abortion group × Years since abortion seeking | 36·83 | 6·19 | 219·16 | 0·0001 |  |
| **Quadratic term (time²)** |  |  |  |  |  |
| Years since abortion seeking² | 0·30 | 0·07 | 1·33 | 0·11 |  |
| Received-abortion group × Years since abortion seeking² | 1·28 | 0·30 | 5·53 | 0·74 |  |
| **Cubic term (time³)** |  |  |  |  |  |
| Years since abortion seeking³ | 1·26 | 1·01 | 1·56 | 0·039 |  |
| Received-abortion group × Years since abortion seeking³ | 0·87 | 0·70 | 1·07 | 0·19 |  |
| **Constant** | 2·52 | 1·66 | 3·80 | <0·0001 |  |
| **Notes:** The number of children born over the five-year period was modeled using mixed-effects Poisson regression, with study group, time since abortion seeking (in years), and their interaction as predictors. Time was modeled using cubic polynomial terms and their interactions with study group to allow for non-linear fertility trajectories. Person-time between interviews (in years) was included as an offset to estimate birth rates. The model included individual-level random intercepts, propensity score weights, and facility-level cluster-robust standard errors. A total of 441 post-index births occurred among 405 women in the *received-abortion* group. The *gave-birth-to-index-pregnancy* group had 275 index births (one per participant) and 23 post-index births among 22 women.  CI = confidence interval. | | | | |  |

| **Supplementary Table 11.** Sensitivity analysis excluding women who miscarried the index pregnancy: Model-predicted birth counts per woman over the five-year period, cumulatively and per 0·5-year intervals, by study group. | | | | | | | | | | |  |
| --- | --- | --- | --- | --- | --- | --- | --- | --- | --- | --- | --- |
|  | **Received-abortion group** | | | **Gave-birth-to-index-pregnancy group** | | |  |  |  |  |  |
|  | Predicted births | Lower CI | Upper CI | Predicted births | Lower CI | Upper CI | Predicted difference in births between study groups | Lower CI | Upper CI | p-value |  |
| **0 - 5 (cumulative)** | 0·37 | 0·28 | 0·45 | 1·04 | 0·97 | 1·12 | 0·68 | 0·55 | 0·80 | <0·0001 |  |
|  |  |  |  |  |  |  |  |  |  |  |  |
| ***Interval (years)*** |  |  |  |  |  |  |  |  |  |  |  |
| 0 - 0·5 | 0·01 | 0·01 | 0·02 | 0·69 | 0·59 | 0·81 | 0·68 | 0·57 | 0·79 | <0·0001 |  |
| 0·5 - 1 | 0·03 | 0·02 | 0·05 | 0·25 | 0·21 | 0·29 | 0·22 | 0·17 | 0·26 | <0·0001 |  |
| 1 - 1·5 | 0·05 | 0·04 | 0·07 | 0·07 | 0·05 | 0·10 | 0·02 | -0·01 | 0·04 | 0·25 |  |
| 1·5 - 2 | 0·06 | 0·05 | 0·08 | 0·02 | 0·01 | 0·05 | -0·04 | -0·07 | -0·02 | <0·0001 |  |
| 2 - 2·5 | 0·06 | 0·05 | 0·07 | 0·005 | 0·001 | 0·03 | -0·06 | -0·07 | -0·04 | <0·0001 |  |
| 2·5 - 3 | 0·05 | 0·04 | 0·06 | 0·002 | 0·0001 | 0·02 | -0·05 | -0·06 | -0·04 | <0·0001 |  |
| 3 - 3·5 | 0·04 | 0·03 | 0·05 | 0·001 | 0·00004 | 0·02 | -0·04 | -0·04 | -0·03 | <0·0001 |  |
| 3·5 - 4 | 0·03 | 0·02 | 0·03 | 0·001 | 0·00003 | 0·02 | -0·03 | -0·03 | -0·02 | <0·0001 |  |
| 4 - 4·5 | 0·02 | 0·02 | 0·03 | 0·002 | 0·0001 | 0·03 | -0·02 | -0·02 | -0·01 | <0·0001 |  |
| 4·5 - 5 | 0·02 | 0·01 | 0·03 | 0·01 | 0·001 | 0·05 | -0·01 | -0·02 | 0·01 | 0·36 |  |
| **Notes:** The number of children born over the five-year period was modeled using mixed-effects Poisson regression, with study group, time since abortion seeking (in years), and their interaction as predictors. Time was modeled using cubic polynomial terms and their interactions with study group to allow for non-linear fertility trajectories. Person-time between interviews (in years) was included as an offset to estimate birth rates. The model included individual-level random intercepts, propensity score weights, and facility-level cluster-robust standard errors. Interval-specific predicted birth counts were obtained by multiplying estimated birth rates by the interval length (0·5 years); cumulative birth counts were calculated by summing predicted counts across all intervals while accounting for their covariance. CI = confidence interval. | | | | | | | | | | |  |
|  |  |  |  |  |  |  |  |  |  |  |  |

| **Supplementary Table 12.** Sensitivity analysis excluding women who miscarried the index pregnancy: Mixed-effects logistic regression estimates and model-predicted probabilities that subsequent births were undesired prior to conception, by study group. | | | | | | | | |
| --- | --- | --- | --- | --- | --- | --- | --- | --- |
| **Panel A:** Adjusted odds that a subsequent birth was undesired (before conception). | | | | | | | | |
|  | **Unweighted model** | | | | **Weighted model** | | | |
| **Predictor** | **AOR** | **Lower CI** | **Upper CI** | **p-value** | **AOR** | **Lower CI** | **Upper CI** | **p-value** |
| Gave-birth-to-index-pregnancy group *(ref.)* | |  |  |  |  |  |  |  |
| Received-abortion group | 0·11 | 0·03 | 0·35 | 0·0002 | 0·10 | 0·03 | 0·32 | 0·0001 |
| Years since abortion seeking | 1·03 | 0·98 | 1·07 | 0·22 | 1·00 | 0·98 | 1·03 | 0·71 |
| Constant | 1·96 | 0·39 | 9·85 | 0·42 | 4·74 | 0·93 | 24·26 | 0·06 |
| **Panel B:** Model-predicted probabilities of a subsequent birth being undesired (before conception). | | | | | | | | |
|  | **Unweighted model** | | | | **Weighted model** | | | |
|  | **Predicted probability** | **Lower CI** | **Upper CI** | **p-value** | **Predicted probability** | **Lower CI** | **Upper CI** | **p-value** |
| Gave-birth-to-index-pregnancy group | 81·8% | 65·4% | 98·1% | - | 85·0% | 70·3% | 99·7% | - |
| Received-abortion group | 36·6% | 31·0% | 42·3% | - | 35·6% | 27·2% | 44·1% | - |
| Difference, percentage points | 45·1 | 29·3 | 60·8 | <0·0001 | 49·4 | 35·1 | 63·7 | <0·0001 |
| **Notes:** Models included study group of the mother, years since mother’s abortion seeking (a continuous variable), and a random intercept for each mother to account for multiple births per individual, with cluster-robust standard errors at the facility level. The weighted model included propensity-score derived weights to balance baseline characteristics across study groups. A birth was classified as undesired if the mother’s most recent pre-pregnancy DAP score was >2·5, indicating a strong desire to avoid pregnancy. Among the 464 subsequent (post-index) births, 441 occurred to 405 women in the *received-abortion* group and 23 occurred to 22 women in the *gave-birth-to-index-pregnancy* group. Pre-pregnancy DAP scores were available for 455 births; nine births (eight in the *received-abortion* group and one in the *gave-birth-to-index pregnancy* group) lacked pre-pregnancy DAP scores and were excluded. DAP = Desire to Avoid Pregnancy scale; AOR = adjusted odds ratio; CI = confidence interval. | | | | | | | | |
